# Identification of microRNA-regulated pathways using an integration of microRNA-mRNA microarray and bioinformatics analysis in CD34+ cells of myelodysplastic syndromes

**DOI:** 10.1038/srep32232

**Published:** 2016-08-30

**Authors:** Feng Xu, Yang Zhu, Qi He, Ling-Yun Wu, Zheng Zhang, Wen-Hui Shi, Li Liu, Chun-Kang Chang, Xiao Li

**Affiliations:** 1 Department of Hematology, Shanghai Jiao Tong University Affiliated Sixth People’s Hospital, Shanghai, China.

## Abstract

The effect of microRNA (miRNA) and targeted mRNA on signal transduction is not fully understood in myelodysplastic syndromes (MDS). Here, we tried to identify the miRNAs-regulated pathways through a combination of miRNA and mRNA microarray in CD34+ cells from MDS patients. We identified 34 differentially expressed miRNAs and 1783 mRNAs in MDS. 25 dysregulated miRNAs and 394 targeted mRNAs were screened by a combination of Pearson’s correlation analysis and software prediction. Pathway analysis showed that several pathways such as Notch, PI3K/Akt might be regulated by those miRNA-mRNAs pairs. Through a combination of Pathway and miRNA-Gene or GO-Network analysis, miRNAs-regulated pathways, such as miR-195-5p/DLL1/Notch signaling pathway, were identified. Further qRT-PCR showed that miR-195-5p was up-regulated while DLL1 was down-regulated in patients with low-grade MDS compared with normal controls. Luciferase assay showed that DLL1 was a direct target of miR-195-5p. Overexpression of miR-195-5p led to increased cell apoptosis and reduced cell growth through inhibition of Notch signaling pathway. In conclusion, alteration expression of miRNAs and targeted mRNAs might have an important impact on cancer-related cellular pathways in MDS. Inhibition of Notch signaling pathway by miR-195-5p-DLL1 axis contributes to the excess apoptosis in low-grade MDS.

Myelodysplastic syndromes (MDS) are a group of clonal diseases that are characterized by the abnormal development of hematopoietic cells and the high risk of evolution to leukemia^1^. The pathogenesis of MDS is poorly understood because of its heterogeneity and complexity^1^.

MicroRNAs (miRNAs) are a cluster of small non-coding RNAs (19–25 nucleotide) that lead to translation inhibition or mRNA degradation via binding to target mRNA untranslated regions (UTRs)^2^. MiRNAs are important regulators of hemopoietic stem/progenitor cell (HSC) function^3^,^4^,^5^,^6^,^7^,^8^,^9^,^10^,^11^,^12^. MiR-125a controls the size of the stem cell population via the regulation of HSC apoptosis^3^. MiR-221, miR-222 and miR-451 regulate erythroid differentiation^4^,^5^. MiR-223 and miR-155 regulate granulocytopoiesis/monocytopoiesis^6^,^7^. MiR-150 and miR-181 regulate the differentiation of B cells and T lymphocytes^8^,^9^. MiR-150, miR-145, miR-146a and miR-34 regulate megakaryocytopoiesis^10^,^11^,^12^. Malignant clonal cells of MDS originate from HSCs, and multi-lineage dysplasia is frequently observed in MDS. In view of the key role of miRNAs in the regulation of hematopoiesis, the association between miRNAs and MDS pathogenesis is worthy of further investigation.

Previous miRNAs-related studies investigated the relationship between miRNAs and target mRNAs using *in vitro* experiments, such as luciferase activity assays. The interaction of microRNA-mRNA in clinical samples should present as a network that is characterized by an miRNA that corresponds to multiple mRNAs, which is difficult to demonstrate in *in vitro* experiments. The pathogenesis-related signaling pathways could be screened by using high throughput bioinformatics analysis based on the miRNA-mRNA network. However, these types of analyses were not performed. This study constructed paired miRNA-mRNAs expression profiles and clusters of miRNA target genes and further identified microRNA-regulated pathways by integrating microarray data and bioinformatics analysis in CD34+ cells of MDS.

## Materials and Methods

### Patients and cells

MDS was diagnosed using the minimum diagnostic criteria (Vienna, 2006)^13^. The classification and prognostic risk scoring of MDS were performed according to the WHO criteria and the revised International Prognostic Scoring System (IPSS-R)^14^,^15^. A total of 36 MDS patients, including 20 males and 16 females, were involved in this study. Their median age was 58 years (29–81 years). Patients were classified as RCMD (n = 17), RAEB-1 (n = 10) and RAEB-2 (n = 9). Supplementary Table 1 shows the patient characteristics. The control group contained a total of 24 healthy volunteers with a median age of 52 years (19–91 years). The ethics committee of the Sixth Hospital affiliated with Shanghai Jiao Tong University approved this research. All subjects provided informed consent in accordance with the Declaration of Helsinki. The methods were carried out in accordance with the approved guidelines. CD34+ cells were isolated using magnetic-activated cell sorting (MACS) from bone marrow mononuclear cells according to the manufacturer’s protocol. K562 and HEK-293T cells were obtained from ATCC. SKM-1 cells were a gift from Prof. Nakagawa^16^. All cell lines were maintained in complete medium (RPMI 1640 supplemented with 10% heat-inactivated fetal bovine serum, 1% glutamine, and 1% sodium pyruvate).

### miRNA and mRNA expression microarray

The were used for the microarray study. The miRNA or mRNA expression profiles of CD34+ cells from 12 MDS patients and 6 normal controls were determined using Affymetrix miRNA 3.0 Expression Array or Primeview Human Gene Expression Array (Affymetrix, US). Clinical characteristics of those patients were shown in Supplementary Table 2. One microgram of total RNA was tailed with poly A and biotin-labeled using the FlashTag Biotin HSR kit (Affymetrix) according to manufacturer’s instructions for miRNA microarrays. One microgram of total RNA was reverse transcribed, amplified and biotin-labeled using the Genechip 3′IVT Express Kit (Affymetrix) according to manufacturer’s instructions for mRNA expression microarrays. The biotin-labeled products were loaded on Genechips for subsequent hybridization. The Genechips were washed and stained in the Fluidics station using a specified protocol. Signal intensities were acquired using a Genechip Scanner 3000 7G (Affymetrix) to generate cell intensity files (CEL). Statistical analysis was performed using Partek Genomics Suite software (Partek Inc., St. Louis, MO, USA). A robust multi-array average (RMA) algorithm was used to normalize the data. The false discovery rate (FDR) was less than 0.1 to minimize the false identification of genes. Changes greater than 1.5-fold were analyzed for up- or down-regulated genes. Hierarchical clustering based on genes and samples was performed using Cluster 3.0 software. The RVM t-test was applied to filter differentially expressed genes in the control and experiment groups because the RVM t-test effectively increases the degrees of freedom in small samples. We performed significance and FDR analyses, and the differentially expressed genes were selected according to the p-value threshold. A *P* value < 0.05 was considered a significant difference.

### GO and Pathway analysis

GO analysis was used to analyze the main function of differently expressed genes according to the Gene Ontology, which is the key functional classification of NCBI. This classification system organizes genes into hierarchical categories and identifies the gene regulatory network based on biological process and molecular function. A two-tailed Fisher’s exact test and Χ^2^ test were used to classify the GO category, and the FDR was calculated to correct the *P* value. A smaller FDR indicated a smaller error in judging the p value. Pathway analysis was used to determine significant pathways based on differential gene expression according to the KEGG. Fisher’s exact test was used to select the significant pathway, and the threshold of significance was defined by the *P* value and FDR.

### MicroRNA-gene-network analysis

An miRNA-gene-network was constructed by quantification of the relationship between miRNAs and differential gene expression values and their interactions in the Sanger miRNA database. The adjacency matrix of MicroRNA and genes A = [a_ij_] was created using the attribute relationships between genes and microRNAs, where a_ij_ represents the weight of the relationship between gene i and MicroRNA j. Circles in the miRNA-Gene-Network represent one edge. The center of the network is represented by a degree. Degree is the contribution of one miRNA to the genes in the vicinity or the contribution of one gene to the miRNAs in the vicinity. The key miRNA and gene in the network always exhibit the biggest degrees.

### Quantitative real-time PCR

The primers for miRNAs, mRNA, GAPDH and U6 used in reverse transcription and real-time PCR were synthesized by Tiangen Inc. (Beijing, China) Total RNA (100 ng) was reverse transcribed using the Taqman MicroRNA Reverse Transcriptase kit according to the manufacturer’s instructions (Applied Biosystems, US). Real-time qRT-PCR was performed using a real-time PCR system (ABI 7900, Applied Biosystems). MiRNAs and targeted mRNA were determined in 36 MDS patients and 24 normal controls. The expression of miRNAs or mRNA was defined from the threshold cycle, and relative expression levels were calculated using the 2−ΔΔCt method after normalization with reference to the expression of U6 small nuclear RNA or GAPDH.

### Lentivirus-mediated overexpression of miR-195-5p

A full miR-195-5p premature sequence was cloned into hU6-MCS-Ubiquitin-EGFP-IRES-puromycin (purchased from JiKai Inc., Shanghai, China). The construction of the miR-195-5p-expressive vector was confirmed using restriction analysis and DNA sequencing. Lentivirus packaging and titer were determined (1–3 × 10^8^ TU/mL), and the phU6-miR-195-5p lentivirus was transfected into SKM-1 and K562 cells. Briefly, 5 × 10^5^ cells/well in a 6-well plate were incubated with the virus and polybrene (6 μg/ml) in a 1 mL volume. Cells with stable expression of the miR-195-5p or a control miRNA were established in the presence of puromycine (10 mg/ml) in medium. The overexpression efficiency of the miR-195-5p was evaluated using qRT-PCR.

### Measurement of cell proliferation, apoptosis, cycle and colony formation

Transfected cells were plated in 96-well plates at a density of 5 × 10^3^ cells/well in quintuplicate. Ten microliters of a WST-1 working solution (Keygen, Nanjing, China) was added to each well, and the cells were incubated for 2 hours. The absorbance at 450 nm was measured using a microplate reader. The inhibition rate of cell proliferation was calculated as follows: % of inhibition rate = each time point (OD^treated well^ − OD^blank well^)/original point (OD^unreated well^ − OD^blank well^). Cell apoptosis was analyzed using FACS and Annexin V–APC. For cell-cycle analysis, 10^5^ cells were washed with cold PBS, fixed in 70% ethanol, washed with PBS, and re-suspended in 1 mL of 7-AAD staining reagent (50 mg/ml propidium iodide and 1 mg/ml RNAse). Samples were incubated in the dark for 30 min before cell cycle analysis. Cell cycle was measured using FACS Calibur. The percentages of cells in the G1, S and G2 phases were calculated using Cellquest software. For colony formation, transfected cells were plated in 12-well plates in Methocult H4434 methylcellulose medium containing SCF, GM-CSF, IL-3, and erythropoietin (StemCell Technologies, Hangzhou, CN) at 2 × 10^3^ cells/well in duplicate wells for each condition. Cells were incubated for 14 days in a humidified incubator at 37 °C, and colonies that contained at least 30 cells were counted.

### pGL3-DLL1 promoter construction and luciferase assay

pGL3-DLL1-3′UTR-Luc vectors contained the 3′-UTR region of the DLL1 transcript. HEK-293T cells were seeded at a density of 2 × 10^5^ cells per well in 6-well plates. Increasing amounts of phU6-miR-195-5p expression constructs (2.5 ug), 1 μg of pGL3-DLL1-Luc or mutant vectors containing firefly luciferase and 0.2 μg of pRL-SV40 vector containing renilla luciferase were co-transfected into 293T cells using Hiperfect transfection reagent according to the manufacturer’s protocol to investigate the target inhibition effect of miR-195-5p on DLL1. Luciferase activities were assayed 24 h after transfection using the DLR™ (Dual Luciferase^®^) Assay in a BioTek Synergy™ 2. Firefly luciferase activity was normalized to Renilla luciferase. Luciferase activity is expressed as relative light units (RLU).

### Flow cytometry analysis of DLL1 protein

Cells were labeled with anti-DLL1-PE at room temperature for 15 minutes. Cells were washed twice, and labeled cells were analyzed using FACS Calibur. DLL1 expression was quantified using the relative mean fluorescence intensity (RMFI) (the mean fluorescence intensity of the antigen staining divided by the mean fluorescence intensity of the isotype-matched negative control staining). DLL1 expression was determined in 5 cases with normal controls, 4 cases with RAEB-2 patients and cell lines including MDS-L, K562, SKM-1 and U937.

### Western blotting

SKM1 and K562 cells (10^7^) were lysed using cell lysis buffer. Total cell extracts were fractionated on 10% sodium dodecyl sulfate polyacrylamide gels, electroblotted to polyvinylidene difluoride membranes (Millipore, Billerica, MA), and reacted with the following primary antibodies: DLL1, DLL3, cleaved Notch1, bcl-2, BAX, β-catenin and GAPDH (1:1000, Cell Signaling Technology). Secondary human anti-rabbit antibodies (1:1500; Cell Signaling Technology) conjugated to horseradish peroxidase were used for enhanced chemoluminescence (Pierce Chemical, Rockford, IL), and the membranes were exposed to film.

### Statistical analysis

All statistical analyses were performed using the SPSS 11.0 System. The comparison of two independent samples was performed using the two-sample *t* test. Multiple pairwise comparisons were performed using one-way analysis of variance (*ANOVA*). Correlations between miRNA and mRNA expression profiles were calculated using Pearson’s correlation. A *P* < 0.05 was considered to be statistically significant.

## Results

### Identification of target-relationship miRNA-mRNA pairs in CD34+ cells of MDS patients

**Figure 1:**
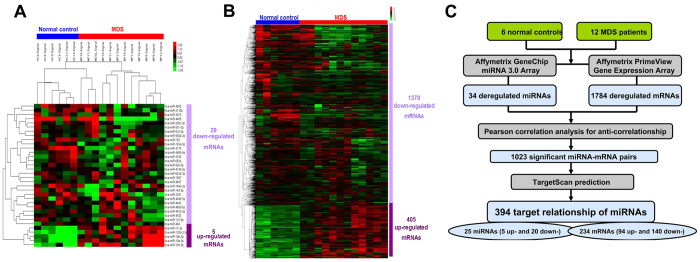
Identification of target-relationship miRNA-mRNA pairs. (**A**) The Affymetrix microRNA microarray revealed differential miRNA expression profiles in MDS. (**B**) The Affymetrix mRNA microarray revealed differential gene expression profiles. (**C**) Multi-step approach was performed to identify target-relationship miRNA-mRNA pairs.

We identified 34 differentially expressed miRNAs compared to normal controls: 5 miRNAs were upregulated, and 29 miRNAs were downregulated (Fig. 1A). The gene expression profiles of the same 18 samples were analyzed using Affymetrix GeneChip PrimeView Array. A total of 1783 mRNAs (405 up- and 1378 down-regulated mRNAs) were significantly differentially expressed in MDS patients compared to normal controls (Fig. 1B). A multi-step approach was adopted to further identify the target relationship of miRNAs-mRNAs (Fig. 1C). Significant anti-correlations between miRNA-mRNA pairs were determined using Pearson’s correlation analysis based on the significantly aberrant expression profiles of miRNA and mRNA in 12 MDS patients and 6 normal controls. Only the target relationship miRNA-mRNA pairs predicted by TargetScan were screened. The results identified 25 dysregulated miRNAs and 234 targeted mRNAs, and 394 target relationships of miRNAs were established.

### Pathway and GO analyses of the miRNA targets

**Figure 2:**
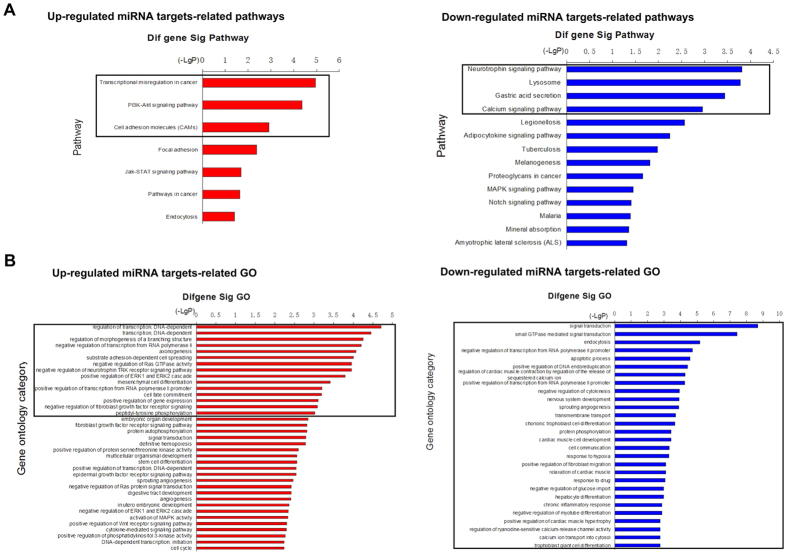
Pathway and GO analyses of the miRNA targets. (**A**) Pathways that were significantly affected by the up-regulated miRNA targets included transcriptional misregulation in cancer, PI3K-Akt signaling pathway and cell adhesion (left panel). The pathways that were significantly affected by the down-regulated miRNA targets included neurotrophin signaling pathway, lysosome, gastric acid secretion and calcium signaling pathway (right panel). (**B**) The GO terms that were significantly affected by the up-regulated miRNA targets included regulation of transcription, cell fate commitment, regulation of Ras GTPase and others (left panel). The GO terms that were significantly affected by the down-regulated miRNA targets included signal transduction, apoptotic process, regulation of transcription and others (right panel). Black Box represented that the FDR value of pathway or GO is less than 0.05.

Pathway analysis demonstrated that 7 pathway categories that were affected by up-regulated miRNA targets and 14 categories that were affected by down-regulated genes were enriched with a p-value < 0.05 (Supplementary Table 3 and Fig. 2A). However, 3 (Transcriptional misregulation in cancer, PI3K-Akt signaling pathway and cell adhesion) and 4 (Neurotrophin signaling pathway, lysosome, gastric acid secretion and calcium signaling pathway) pathway categories were significantly enriched using a cutoff of FDR < 0.05. GO analysis revealed that 210 significant GO categories (p < 0.05) were regulated by up-regulated miRNA targets and 244 significant categories were regulated by down-regulated miRNA targets (Supplementary Table 4 and Fig. 2B). Fifteen GO categories (e.g., regulation of transcription, cell fate commitment, and regulation of Ras GTPase) and 28 (e.g., signal transduction, apoptotic process, and transcription regulation) GO categories were significantly enriched using a cutoff of FDR < 0.05. We also identified 42 key miRNA targets that were shared in significant Pathway and GO categories, including HMGA2, KLF3, FGFR2, EFNA1, SPRY1/2, c-KIT, CCND2, IGF2R, DLL1, TNF, CAMK2D, and CDC25B (Supplementary Fig. 1 and Supplementary Table 5). Some of these genes, such as HMGA2 and c-KIT, were reported in previous studies, which affirmed their important roles in the pathogenesis of MDS^17^,^18^.

### Identification of miRNA-regulated pathways based on miRNA-gene- Network and Go-Network analyses

We performed miRNA-gene-Network and miRNA-Go-Network analyses of the screened miRNA targets. The results demonstrated that miR-19a/b-3p, miR-195-5p, miR-145-5p, miR-148a-3p, miR-200c-3p, and miR-17-3p were considered regulators in the miRNA-gene-Network because the number of their targets was over 3 (Supplementary Fig. 1). Notably, miR-19a/b-3p and miR-195-5p possessed over 10 targeted genes, and these miRNAs may perform an important role in the regulation of gene expression in MDS. miRNA-Go-Network analysis revealed similar results that miR-19a/b-3p, miR-195-5p, miR-145-5p, miR-148a-3p and miR-17-3p regulated numerous GO categories (over 60 biological processes) (Supplementary Fig. 2). These analyses identified 29 miRNA-mRNA-regulated pathways, including the miR-195-5p/DLL1/Notch signaling pathway, miR-148a/TEK/PI3K-Akt signaling pathway, miR-195-5p/BDNF/MAPK signaling pathway, and miR-145/CCND2/JAK-STAT signaling pathway (Table 1).

### Validation of miRNAs and mRNAs expression levels using qRT-PCR

**Figure 3:**
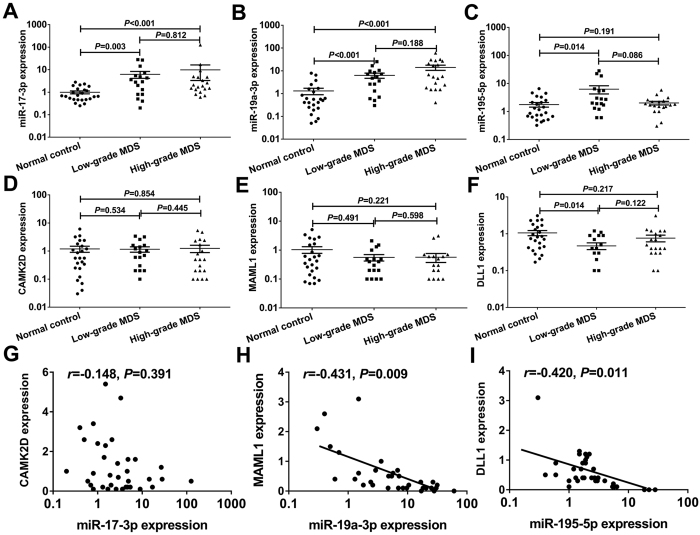
Validation of screened miRNAs and mRNAs using qRT-PCR. The expression of miR-17-3p (**A**) and miR-19a-3p (**B**) were significantly higher in the high-grade and low-grade MDS groups than those in the normal control group (all *P* < 0.05). (**C**) The patients with low-grade MDS exhibited significantly higher miR-195-5p expression than the normal controls (*P* = 0.014). (**D**,**E**) No significant difference in CAMK2D and MAML1 expression was observed between the normal controls and MDS group. (**F**) DLL1 expression was significantly lower in the low-grade compared to the normal controls (*P* = 0.014). (**G**) No significant anti-correlation was observed between miR-17-3p and CAMK2D expression. (**H**,**I**) miR-19a-3p and miR-195-5p expression exhibited anti-correlations with MAML1 and DLL1 expression, respectively (*r* = −0.431, *P* = 0.009; *r* = −0.420, *P* = 0.011). Error bars throughout represent the s.e.m. Comparison analysis was performed using one-way ANOVA HSD test. Correlation coefficient was calculated using Spearman analysis. The detection of miRNAs or mRNAs using quantitative RT-PCR was replicated three times.

We used qRT-PCR to determine the expression levels of miRNA and mRNA in CD34+ cells from an extended cohort of MDS patients to validate the miRNAs and targeted mRNAs that were identified in the microarray and bioinformatics analyses. The primer sequences for qRT-PCR were shown in Supplementary Table 6. Data from miRNAs and mRNAs expression analyses was shown in Supplementary Table 7. The expression of miR-17-3p and miR-19a-3p in CD34+ cells was significantly higher in the low-grade and high-grade MDS group compared to the normal controls (all *P* < 0.05) (Fig. 3A,B). The expression of miR-195-5p was higher in the low-grade MDS group compared to the normal controls (*P* = 0.014), and no difference was observed between the low-grade and high-grade MDS group (*P* = 0.086) (Fig. 3C). The expression of the mRNAs targeted by these miRNAs, including CAMK2D, DLL1 and MAML1, was also determined, and correlations with miRNAs were evaluated. QRT-PCR revealed no significant differences in CAMK2D expression between the normal controls and MDS group (Fig. 3D). Similarly, no significant differences were observed in CAMK2D expression between the normal controls and MDS group (Fig. 3E). In contrast to miR-195-5p expression, DLL1 expression was significantly lower in the low-grade MDS group compared to the normal controls (*P* = 0.014) (Fig. 3F). No significant anti-correlation was observed between miR-17-3p and CAMK2D expression (Fig. 3G). However, miR-19a-3p and miR-195-5p expression exhibited anti-correlations with MAML1 and DLL1 expression, respectively (*r* = −0.431, *P* = 0.009; *r* = −0.420, *P* = 0.011) (Fig. 3H,I).

### miR-195-5p post-transcriptionally reduces DLL1 expression by directly targeting its 3′UTR

**Figure 4:**
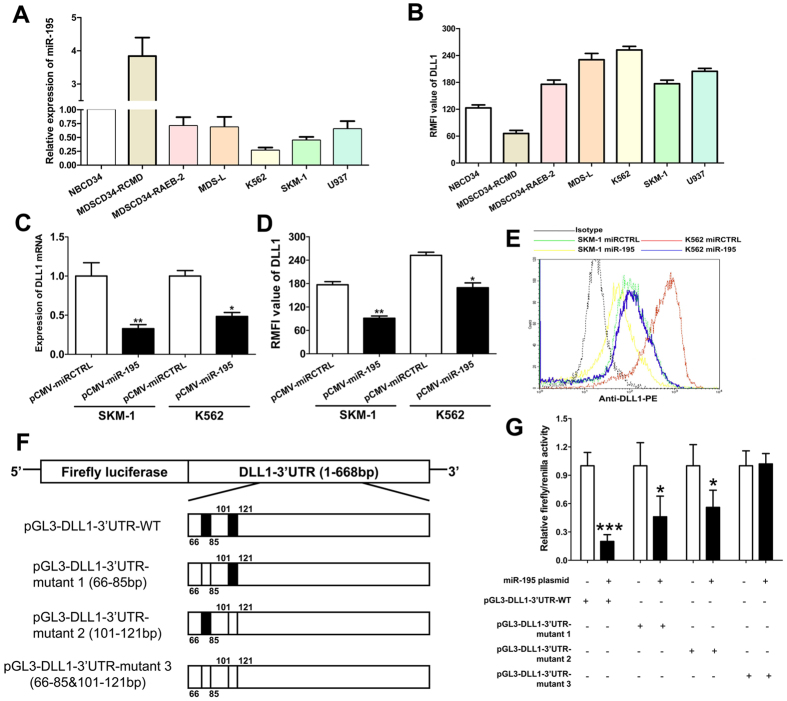
miR-195-5p reduces DLL1 expression by directly targeting its 3′UTR. (**A**) miR-195-5p was up-regulated in patients with MDS-RCMD and down-regulated in the K562 and SKM-1 cell lines. (**B**) DLL1 was down-regulated in patients with MDS-RCMD and up-regulated in the K562 and SKM-1 cell lines. (**C**,**D**) Overexpression of miR-195-5p significantly decreases the expression of DLL1 mRNA and protein in SKM-1 and K562 cells. (**E**) The representative flow graphic for DLL1 detection is shown. (**F**) A series of 3′UTR fragments of DLL1, including full length, binding site 1, binding site 2, and their corresponding mutant counterparts, were directly fused downstream of the firefly luciferase gene. (**G**) miR-195-5p post-transcriptionally reduces DLL1 expression by directly targeting its 3′UTR. Error bars throughout represent the s.e.m. *P < 0.05; **P < 0.01 and ***P < 0.001. Unpaired Student’s t tests were used to calculate the *P* value in comparison analyses. All experiments were replicated three times.

We performed a series of functional experiments to investigate the targeted regulation of miR-195-5p on DLL1. We screened miR-195-5p and DLL1 expression in several MDS/leukemia cell lines. QRT-PCR analysis demonstrated that miR-195-5p was down-regulated, and DLL1 was up-regulated in the MDS/leukemia cell lines (Fig. 4A,B). Therefore, we used SKM-1 and K562 cells to perform a series of functional experiments. We constructed SKM-1 and K562 cells to stably overexpress miR-195-5p using a lentivirus-mediated transfection system. The expression of DLL1 mRNA and protein were significantly reduced in SKM-1 and K562 cells with miR-195-5p overexpression compared to the control cells (Fig. 4C,D). Figure 4E shows the representative flow graphic for DLL1 detection. A series of 3′UTR fragments of DLL1, including the full length, binding site 1, binding site 2, and their corresponding mutant counterparts, were directly fused downstream of the firefly luciferase gene to investigate whether DLL1 was a direct target of miR-195-5p (Fig. 4F). HEK-293T was used to perform luciferase experiments because of stability and high efficacy in transfection experiments. 293T cells have lower expression of miR-195-5p than MDS cells (Supplementary Figure 3). Figure 4G show that miR-195-5p decreased the relative luciferase activity of the full length DLL1-3′-UTR construct, but luciferase activity was not significantly altered in the construct with both sites mutated. Our results also demonstrated that miR-195-5p targeted each of the binding sites of the DLL1 3′-UTR. Luciferase activity was reduced when one of the 2 sites was mutated compared to activity when both sites were mutated. Taken together, these results indicate that miR-195-5p post-transcriptionally reduced DLL1 expression by directly targeting its 3′UTR.

### Overexpression of miR-195-5p elevated cell apoptosis and reduced cell growth

**Figure 5:**
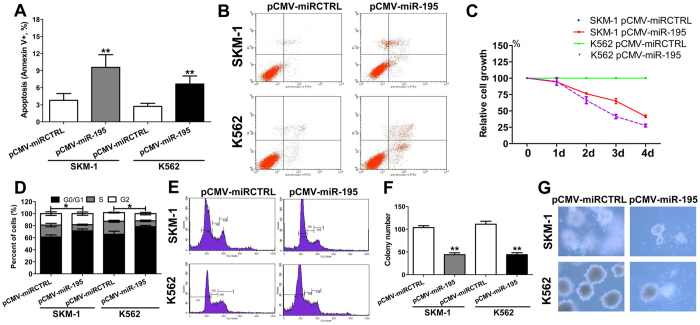
Overexpression of miR-195-5p results in an elevated cell apoptosis and reduced cell growth. (**A**) Overexpression of miR-195-5p significantly increased cell apoptosis in SKM-1 and K562 cells; (**B**) The representative flow graphic for apoptosis detection in the cells transfected with pCMV-miR-195-5p or control vector is shown; (**C**) Overexpression of miR-195-5p resulted in significant growth inhibition of SKM-1 and K562 cells; (**D**) Overexpression of miR-195-5p induced an incremental decrease in the percentage of cells in S phase and cell cycle arrest in the G0/G1 phase; (**E**) The representative flow graphic for cell cycle detection is shown; (**F**) Overexpression of miR-195-5p significantly decreased colony formation; (**G**) The representative graphic for colony detection is shown. Error bars throughout represent the s.e.m. *P < 0.05; **P < 0.01 and ***P < 0.001 relative to the pCMV control vector. Throughout the figure, unpaired Student’s t tests were used to calculate all *P* values. The data shown in the graphics are representative values from three independent experiments.

We also investigated the effect of miR-195-5p on malignant phenotypes of MDS/leukemia cell lines. Notably, overexpression of miR-195 increased cell apoptosis in SKM-1 and K562 cells (Fig. 5A). Figure 5B shows the representative flow graphic for apoptosis detection. Overexpression of miR-195-5p also produced an inhibitory effect on the proliferation of SKM-1 and K562 cells (Fig. 5C). Overexpression of miR-195-5p also induced an incremental increase in the percentage of cells in the G0/G1 phase and a decrease in the percentage of cells in the S phase in SKM-1 and K562 cells (Fig. 5D). Figure 5E shows the representative flow graphic for cell cycle detection. The overexpression of miR-195-5p remarkably suppressed colony formation in these cell lines (Fig. 5F). Figure 5G shows the representative graphic for colony detection. Taken together, our results suggest that miR-195-5p exerts a tumor suppressor function in MDS.

### Overexpression of miR-195-5p inhibited activation of the Notch signaling pathway

**Figure 6:**
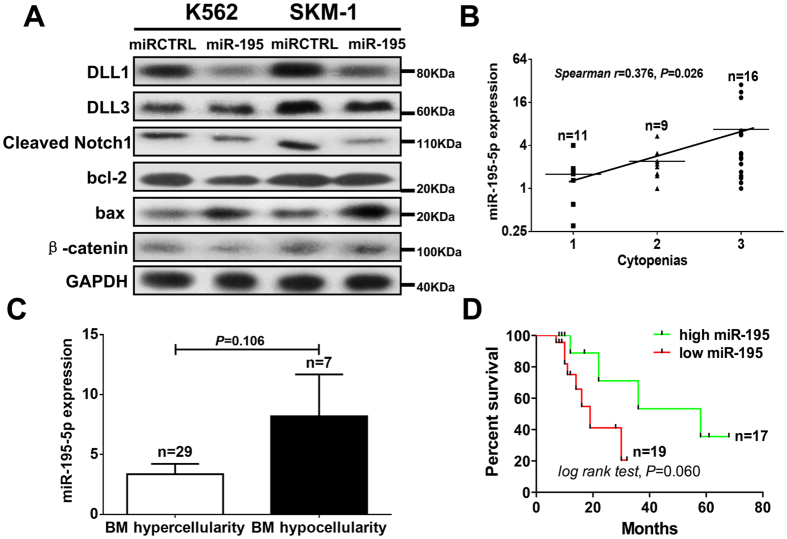
Overexpression of miR-195-5p inhibited the activation of Notch signaling pathway and is associated with severe cytopenias, bone marrow hypocellularity and superior survival. (**A**) Overexpression of miR-195-5p downregulated the expression of Notch signaling-related proteins, including DLL1 and cleaved Notch1. Overexpression of miR-195-5p increased the expression of the apoptosis-related protein BAX and decreased the expression of proliferation-related proteins bcl-2. (**B**) The number of peripheral cytopenias exhibited positive correlation with miR-195-5p expression in MDS patients (*Spearman r* = 0.376, *P* = 0.026). (**C**) The patients with bone marrow hypocellularity also exhibited reduced expression of miR-195-5p compared to patients with hypercellularity. (**D**) The patients with high expression of miR-195-5p exhibited relatively superior overall survival compared with patients with a low expression of miR-195-5p, but no significant difference was observed. A representative Western blot graphic is shown from three independent experiments. Error bars throughout represent the s.e.m. Correlation coefficient was calculated using Spearman analysis. In comparison analysis, unpaired Student’s t tests were used to calculate the *P* value. Statistical significance in survival analysis was determined using the log-rank test.

DLL1 is a critical Notch signaling ligand that binds the Notch receptor and activates the Notch signaling pathway. We further investigated the mechanism of miR-195-5p-induced cell apoptosis via the Notch signaling pathway. Cleaved Notch1 is a critical activator of Notch signaling. The overexpression of miR-195-5p downregulated the expression of DLL1 and cleaved Notch1 in SKM-1 and K562 cells (Fig. 6A). The overexpression of miR-195-5p increased the expression of the apoptosis-related protein BAX, while decreased the expression of proliferation-related proteins bcl-2 (Fig. 6A). The overexpression of miR-195-5p also inhibited the expression of β-catenin in K562 cells. Our data demonstrated that the overexpression of miR-195-5p induced cell apoptosis via the inhibition of Notch signaling pathway activation.

### Overexpression of miR-195-5p is associated with severe cytopenias, bone marrow hypocellularity and superior survival

The number of peripheral cytopenias exhibited a positive correlation with miR-195-5p expression in MDS patients (*Spearman r* = 0.376, *P* = 0.026) (Fig. 6B). Patients with bone marrow hypocellularity exhibited increased miR-195-5p expression compared to patients with hypercellularity although no difference was observed (Fig. 6C). It seemed that high miR-195-5p expression predicted bone marrow failure. Patients with high miR-195-5p expression seem to exhibit superior overall survival compared to those with low miR-195-5p expression, but no significant difference was observed (Fig. 6D). These results suggested that the overexpression of miR-195-5p contributed to bone marrow failure in low-grade MDS patients, which is consistent with the pro-apoptotic function of miR-195-5p.

## Discussion

Gene Chip array is a powerful tool for the accurate quantification of the expression levels of miRNAs and mRNAs. Single miRNAs or mRNA microarrays produce massive candidate data, and it is difficult for investigators to screen and identify valuable information. Bioinformatics analyses based on biological data provides greater convenience in these situations. The integration of miRNA-mRNA microarray and bioinformatics analysis revealed some reliable miRNA-mRNA pairs and the pathways that were regulated by these pairs. The narrowed number of miRNAs or mRNA and the targeted study after bioinformatics analysis contributed to the further investigation of the pathogenesis of MDS. We also assessed each pathway in the pathway network using bioinformatics analyses. This study used a multi-step approach to identify miRNA-regulated mRNAs and pathways in MDS. Firstly, Gene Chip arrays were used to screen the expression profiles of miRNAs and mRNAs in 12 MDS cases and 6 normal controls. Secondly, all miRNAs and mRNAs with an anti-correlation relationship were captured using Pearson’s correlation analysis. Thirdly, the reliability of the identified target-relationship pairs of miRNAs-mRNAs was assessed and confirmed using the TargetScan database. Finally, target relationships of miRNAs in MDS were identified. These target relationships and pathways and GO analyses were also used to identify several pathways that were regulated by miRNA-mRNA pairs, including MAPK, Notch, JAK/STAT, and PI3K/Akt signaling pathways. We validated several miRNA-mRNA pairs using qRT-PCR and investigated the role of miR-195-5p/DLL1/Notch signaling in MDS *in vitro*. To our knowledge, this study is the first report using an integration of dual microarrays and bioinformatics analyses in MDS, and the results revealed multiple novel miRNA-mRNA pairs and associated pathways.

MiR-195-5p plays a central role in miRNA-gene-networks and miRNA-GO-networks. Therefore, we validated the expression of miR-195-5p and the target gene DLL1 and investigated the function of miR-195-5p/DLL in MDS. qRT-PCR analyses demonstrated that miR-195-5p expression was up-regulated in patients with low-grade MDS compared to normal controls. Hematopoietic cells in low-grade MDS patients exhibit ineffective hematopoiesis and cell senescence, which are characterized by increased cell apoptosis^19^. Therefore, we hypothesized that the overexpression of miR-195-5p would contribute to the increased cell apoptosis. Our *in vitro* experiments demonstrated that miR-195-5p overexpression promoted cell apoptosis and suppressed cell proliferation. Several previous studies revealed reduced miR-195-5p expression in many types of solid cancers, such as gastric, breast, and colon cancers^20^,^21^,^22^. The overexpression of miR-195-5p in these cancers may inhibit cancer cell proliferation, migration and invasion. These findings indicated that miR-195-5p functioned as a tumor suppressor. The overactivation of tumor suppressor genes, such as TP53, leads to excess apoptosis of erythroid cells in MDS, which was identified as a critical contributor of a special subtype of MDS, 5q syndromes^23^,^24^. Therefore, the overexpression of miR-195-5p may account for excess apoptosis in low-grade MDS. Further clinical analyses also demonstrated that miR-195-5p overexpression was associated with severe cytopenias and bone marrow hypocellularity, which supports our conclusion and provides corroborative evidence.

In high-grade MDS, rapid proliferation and apoptotic resistance drive disease progression to AML. MiR-195-5p expression as a tumor suppressor is relative low in high-grade MDS than in low-grade MDS. It may be speculated that some emerging factors in high-grade MDS such as epigenetic or genetic factors may inhibit the expression of miR-195-5p to overcome excess apoptosis and promote disease progression. In addition, high-grade MDS and leukemia frequently harbor 17p deletion (miR-195-5p locates at 17p13)^25^, which may also explain why miR-195-5p levels are low in high-grade MDS. Further studies should be developed to address this issue.

MiR-195-5p exerted its functions via the downregulation of DLL1 expression in this study. DLL1 is a Notch ligand that is expressed in multiple hematopoietic cells^26^. The activation of Notch signaling promotes proliferation, self-renewal and maintenance of HSCs^27^,^28^. The downregulation of DLL1 resulted in an inhibition of Notch signaling in low-grade MDS and further increased cell apoptosis. The suppression of Notch signaling in low-grade MDS should be considered as a critical contributor of bone marrow failure.

In conclusion, the altered expression of miRNAs and targeted mRNAs may affect cancer-related cellular pathways. Inhibition of the Notch signaling pathway by the miR-195-5p-DLL1 axis contributes to the excess apoptosis in low-grade MDS.

## Additional Information

**Accession codes:** Gene and miRNA expression arrays data have been deposited in the NCBI Gene Expression Omnibus under GEO accession number GSE81173 and GSE81372.

**How to cite this article**: Xu, F. *et al*. Identification of microRNA-regulated pathways using an integration of microRNA-mRNA microarray and bioinformatics analysis in CD34+ cells of myelodysplastic syndromes. *Sci. Rep.* **6**, 32232; doi: 10.1038/srep32232 (2016).

## Supplementary Material

Supplementary Information

## Figures and Tables

**Table 1:** Dysregulated miRNAs, genes and targeted pathways in MDS.

miRNA	Style	Fold change (MDS/control)	Gene symbol	Pathway
miR-148a	Down	−7.70	TEK	PI3K-Akt signaling pathway
			ITGA9	PI3K-Akt signaling pathway
			KIT	PI3K-Akt signaling pathway
			HMGA2	Transcriptional misregulation in cancer
miR-145	Down	−2.96	HHEX	Transcriptional misregulation in cancer
			MEIS1	Transcriptional misregulation in cancer
miR-200c	Down	−2.82	EFNA1	PI3K-Akt signaling pathway
			KLF3	Transcriptional misregulation in cancer
miR-195	Up	6.04	BDNF	MAPK signaling pathway
			CDC25B	MAPK signaling pathway
			DLL1	Notch signaling pathway
			MRAS	MAPK signaling pathway
miR-17	Up	3.32	CAMK2D	Calcium signaling pathway
miR-19a	Up	5.85	MAML1	Notch signaling pathway
			SLC8A1	Calcium signaling pathway
			THBS1	Proteoglycans in cancer
			TNF	MAPK signaling pathway
			TNFRSF1B	Adipocytokine signaling pathway
			ACSL1	Adipocytokine signaling pathway
			EDNRB	Calcium signaling pathway
miR-19b	Up	2.53	CALM1	Calcium signaling pathway
			TNF	Proteoglycans in cancer
