# Supplementary material for: Identification of microRNA-regulated pathways using an integration of microRNA-mRNA microarray and bioinformatics analysis in CD34+ cells of myelodysplastic syndromes
Source: Sci Rep. 2016 Aug 30;6:32232. doi: 10.1038/srep32232 (PMC5004188; doi:10.1038/srep32232)
Supplement: Supplementary Information [file srep32232-s1.doc]

**Identification of microRNA-regulated pathways using an integration of microRNA-mRNA microarray and bioinformatics analysis in CD34+ cells of myelodysplastic syndromes**

Feng Xu^1,2^, Yang Zhu^1,2^, Qi He^1^, Ling-Yun Wu^1^, Zheng Zhang^1^, Wen-Hui Shi^1^, Li Liu^1^, Chun-Kang Chang^1^, Xiao Li^1,*^

^1^Department of Hematology, Shanghai Jiao Tong University Affiliated Sixth People's Hospital

^2^These authors have equal contribution to this work

*Correspondence to: Li Xiao, MD, PhD, Dept. of Hematology, Shanghai Jiao Tong University Affiliated Sixth Hospital, Shanghai, China, 200233. E-mail: lixiao3326@163.com, Tel: +86-021-24058745, Fax: +86-021-64701361

**
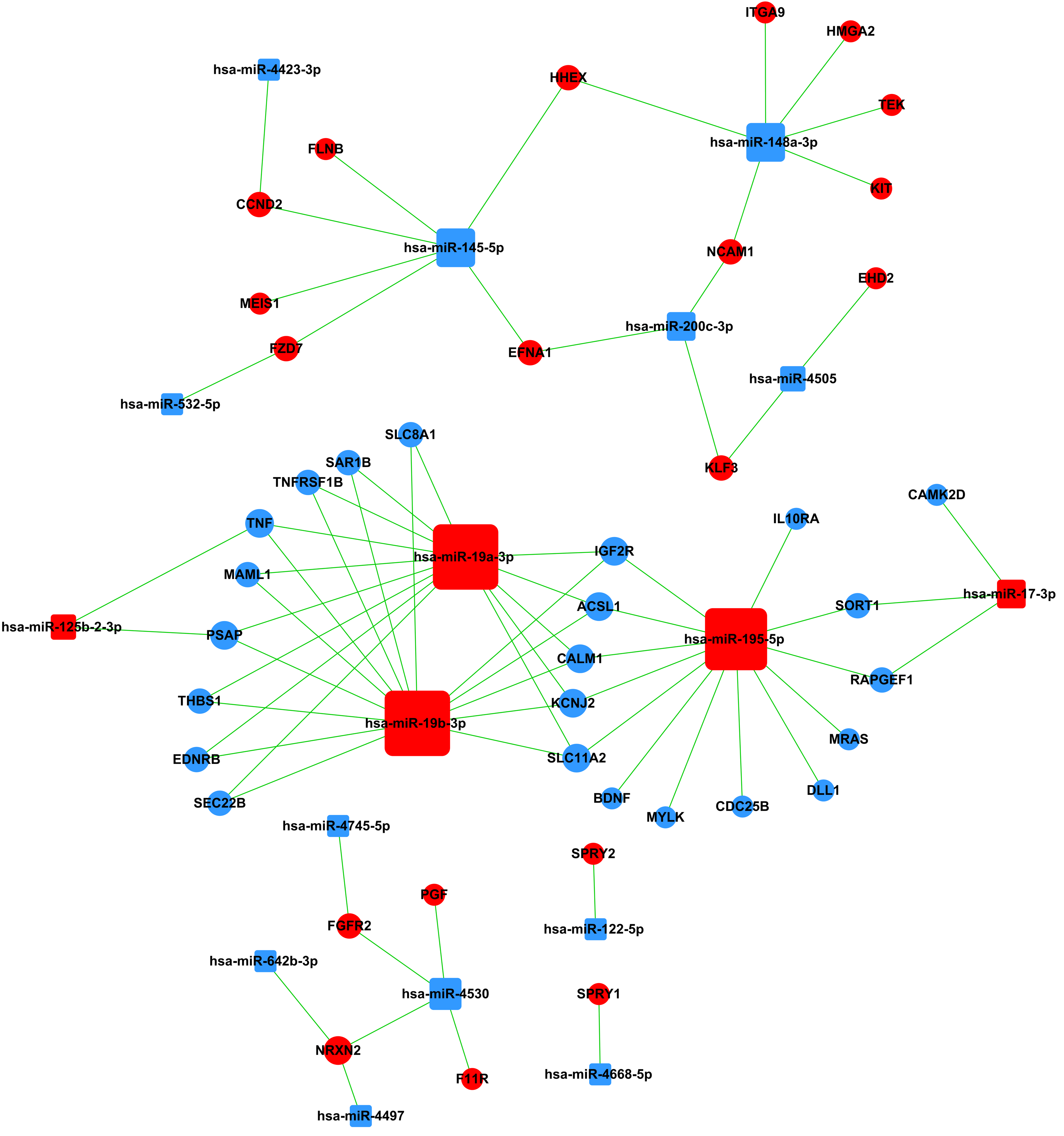
**

**Supplementary Figure 1 miRNA-gene-Network analysis.** miR-19a/b-3p, miR-195-5p, miR-145-5p, miR-148a-3p, miR-200c-3p, and miR-17-3p were considered as regulators in the miRNA-gene-Network, due to the number of their targets over 3.

**
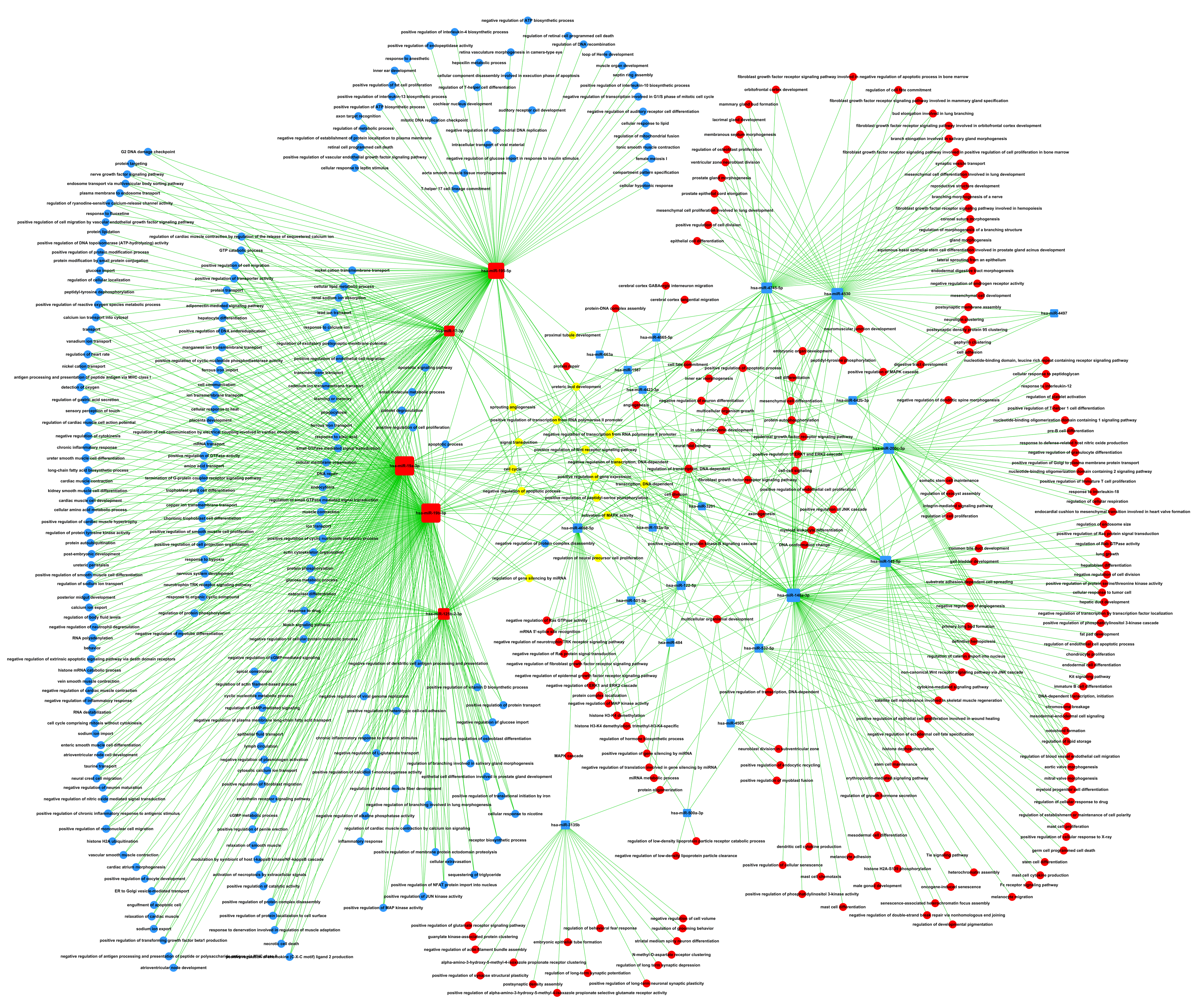
**

**Supplementary Figure 2. miRNA-GO-Network analysis.** miR-19a/b-3p, miR-195-5p, miR-145-5p, miR-148a-3p and miR-17-3p were considered as central regulators in GO-network due to their regulation of numerous GO categories (over 60 biological processes).

**Supplementary Figure 3. Analysis of miR-195 expression in 293T cell line.** 293T cells showed lower expression than CD34+ cells from RAEB-2 or RCMD.

**Supplementary Table 1. Clinical characteristics of 38 patients** with MDS

| **Characteristic** | **Value** |
| --- | --- |
| **Age, y median (range)** | 58 (29-81) |
| **Male: female, n (%)** | 20:16 |
| **Diagnosis, n (%)** | 36 |
| RCMD | 17 (47.2%) |
| RAEB-1 | 10 (27.8%) |
| RAEB-2 | 9 (25.0%) |
| **Karyotype** |  |
| normal | 14 (38.9%) |
| -5/5q- | 4 (11.1%) |
| -7/7q- | 2 (5.6%) |
| +8 | 11 (30.6%) |
| -20/20q- | 1 (2.8%) |
| Complex | 3 (8.3%) |
| others | 1 (2.8%) |
| **Blast percentage in marrow, %** |  |
| <2% | 11 (30.6%) |
| 2-5% | 6 (16.7%) |
| 5-10% | 10 (27.7%) |
| ≥10% | 9 (25.0%) |
| **IPSS-R** |  |
| Lower risk | 16 (44.4%) |
| Higher risk | 20 (55.6%) |
| **Marrow cellularity** |  |
| Hypo | 7 (19.4%) |
| Hyper | 29 (80.6%) |

**Supplementary Table 2. Clinical characteristics of 12 MDS patients involved into mRNA and miRNA array.**

| **No.** | **Sex** | **Age** | **Diagnosis** | **IPSS-R** | **Chr** | **Blast (%)** | **CD34+ (%)** | **Hyperplasity** | **WBC (10^9/L)** | **Neut (%)** | **Hb (g/L)** | **PLT (10^9/L)** |
| --- | --- | --- | --- | --- | --- | --- | --- | --- | --- | --- | --- | --- |
| MP1 | F | 62 | RAEB-1 | 6.5 | Del(9q), Del(20q) | 5.0 | 3.7 | hyper | 2.8 | 68 | 60 | 96 |
| MP2 | M | 45 | RCMD | 4.0 | Tri8 | 3.0 | 1.4 | hyper | 2.4 | 41 | 86 | 20 |
| MP8 | M | 60 | RAEB-1 | 3.0 | Normal | 5.5 | 6.5 | hyper | 1.7 | 60 | 114 | 23 |
| MP9 | M | 68 | RAEB-2 | 7.5 | complex | 10.5 | 11.7 | hyper | 7.7 | 62 | 58 | 61 |
| MP12 | M | 60 | RCMD | 2.0 | Normal | 1.0 | 0.9 | hyper | 2.3 | 29 | 53 | 29 |
| MP14 | M | 61 | RAEB-1 | 3.0 | Normal | 6.5 | 2.9 | hyper | 8.9 | 57 | 79 | 120 |
| MP15 | F | 58 | RCMD | 3.0 | Tri8 | 1.0 | 1.1 | hyper | 1.9 | 55 | 51 | 198 |
| MP16 | M | 52 | RCMD | 6.0 | complex | 3.0 | 3.9 | hyper | 2.7 | 52 | 58 | 64 |
| MP17 | M | 70 | RAEB-1 | 4.5 | Tri8 | 5.0 | 8.7 | hyper | 3.3 | 29 | 130 | 77 |
| MP19 | F | 64 | RAEB-2 | 3.5 | Normal | 11.0 | 12.1 | hyper | 13.0 | 29 | 62 | 342 |
| MP21 | F | 61 | RAEB-2 | 6.0 | Del(5q) | 14.5 | 15.4 | hyper | 2.1 | 30 | 64 | 10 |
| MDSL | M | NA | RAEB-2 | NA | complex | NA | 27.9 | NA | NA | NA | NA | NA |

*NA, not available.

**Supplementary Table 3 Significant pathways affected by miRNAs targets**

| **path_id** | **path_name** | **path_diff gene_count** | **path_gene_count** | **enrichment** | **P value** | **FDR** |
| --- | --- | --- | --- | --- | --- | --- |
| **Significant pathways affected by up-regulated miRNAs targets** | | | | | | |
| 05202 | Transcriptional misregulation in cancer | 6 | 180 | 15.39263158 | 1.12736E-05 | 0.00075533 |
| 04151 | PI3K-Akt signaling pathway | 7 | 347 | 9.315425451 | 4.43949E-05 | 0.001487228 |
| 04514 | Cell adhesion molecules (CAMs) | 4 | 146 | 12.65147801 | 0.001185109 | 0.026467423 |
| 04510 | Focal adhesion | 4 | 206 | 8.966581502 | 0.004307223 | 0.072145977 |
| 04630 | Jak-STAT signaling pathway | 3 | 158 | 8.767954697 | 0.019917733 | 0.253237613 |
| 05200 | Pathways in cancer | 4 | 327 | 5.648672139 | 0.022677995 | 0.253237613 |
| 04144 | Endocytosis | 3 | 204 | 6.790866873 | 0.040095444 | 0.383770676 |
| **Significant pathways affected by dwon-regulated miRNAs targets** | | | | | | |
| 04722 | Neurotrophin signaling pathway | 5 | 120 | 13.44025735 | 0.000152325 | 0.009974203 |
| 04142 | Lysosome | 5 | 122 | 13.21992527 | 0.000164863 | 0.009974203 |
| 04971 | Gastric acid secretion | 4 | 75 | 17.20352941 | 0.000362201 | 0.014608777 |
| 04020 | Calcium signaling pathway | 5 | 183 | 8.81328351 | 0.001107169 | 0.033491852 |
| 05134 | Legionellosis | 3 | 55 | 17.59451872 | 0.002717942 | 0.065774205 |
| 04920 | Adipocytokine signaling pathway | 3 | 71 | 13.62955675 | 0.005711139 | 0.115174642 |
| 05152 | Tuberculosis | 4 | 184 | 7.012308184 | 0.010620502 | 0.183582968 |
| 04916 | Melanogenesis | 3 | 101 | 9.581173559 | 0.015565018 | 0.235420898 |
| 05205 | Proteoglycans in cancer | 4 | 227 | 5.683985488 | 0.022360192 | 0.300620355 |
| 04010 | MAPK signaling pathway | 4 | 260 | 4.962556561 | 0.03572147 | 0.410060986 |
| 04330 | Notch signaling pathway | 2 | 48 | 13.44025735 | 0.039213105 | 0.410060986 |
| 05144 | Malaria | 2 | 49 | 13.16596639 | 0.040799416 | 0.410060986 |
| 04978 | Mineral absorption | 2 | 51 | 12.64965398 | 0.044056139 | 0.410060986 |
| 05014 | Amyotrophic lateral sclerosis (ALS) | 2 | 54 | 11.94689542 | 0.04914869 | 0.424785107 |

**Supplementary Table 4 Significant GO affected by miRNAs targets**

| **path_id** | **path_name** | **path_diff gene_count** | **path_gene_count** | **enrichment** | **P value** | **FDR** |
| --- | --- | --- | --- | --- | --- | --- |
| **Significant GO affected by up-regulated miRNAs targets** | | | | | | |
| GO:0006355 | regulation of transcription, DNA-dependent | 13 | 1304 | 4.603624475 | 1.9439E-05 | 0.00863434 |
| GO:0006351 | transcription, DNA-dependent | 15 | 1827 | 3.791288566 | 3.55382E-05 | 0.00863434 |
| GO:0060688 | regulation of morphogenesis of a branching structure | 2 | 3 | 307.8526316 | 5.56049E-05 | 0.00863434 |
| GO:0000122 | negative regulation of transcription from RNA polymerase II promoter | 8 | 509 | 7.257822355 | 6.25004E-05 | 0.00863434 |
| GO:0007409 | axonogenesis | 4 | 74 | 24.96102418 | 8.45547E-05 | 0.00863434 |
| GO:0034446 | substrate adhesion-dependent cell spreading | 3 | 26 | 53.28218623 | 9.86768E-05 | 0.00863434 |
| GO:0034261 | negative regulation of Ras GTPase activity | 2 | 4 | 230.8894737 | 0.000111053 | 0.00863434 |
| GO:0051387 | negative regulation of neurotrophin TRK receptor signaling pathway | 2 | 4 | 230.8894737 | 0.000111053 | 0.00863434 |
| GO:0070374 | positive regulation of ERK1 and ERK2 cascade | 4 | 87 | 21.23121597 | 0.000160091 | 0.011064074 |
| GO:0048762 | mesenchymal cell differentiation | 2 | 7 | 131.9368421 | 0.000387039 | 0.024073836 |
| GO:0045944 | positive regulation of transcription from RNA polymerase II promoter | 8 | 708 | 5.217841213 | 0.00062668 | 0.032870004 |
| GO:0045165 | cell fate commitment | 3 | 48 | 28.86118421 | 0.000634148 | 0.032870004 |
| GO:0010628 | positive regulation of gene expression | 4 | 131 | 14.10012053 | 0.000783606 | 0.03669184 |
| GO:0040037 | negative regulation of fibroblast growth factor receptor signaling pathway | 2 | 10 | 92.35578947 | 0.000825861 | 0.03669184 |
| GO:0018108 | peptidyl-tyrosine phosphorylation | 3 | 55 | 25.18794258 | 0.000951394 | 0.039451148 |
| GO:0048568 | embryonic organ development | 2 | 13 | 71.04291498 | 0.001425441 | 0.051646769 |
| GO:0008543 | fibroblast growth factor receptor signaling pathway | 4 | 156 | 11.84048583 | 0.001523523 | 0.051646769 |
| GO:0046777 | protein autophosphorylation | 4 | 156 | 11.84048583 | 0.001523523 | 0.051646769 |
| GO:0007165 | signal transduction | 9 | 1030 | 4.034961676 | 0.001621448 | 0.051646769 |
| GO:0060216 | definitive hemopoiesis | 2 | 14 | 65.96842105 | 0.001660668 | 0.051646769 |
| GO:0071902 | positive regulation of protein serine/threonine kinase activity | 2 | 17 | 54.32693498 | 0.002471391 | 0.071912931 |
| GO:0007275 | multicellular organismal development | 6 | 488 | 5.677610009 | 0.002771581 | 0.071912931 |
| GO:0048863 | stem cell differentiation | 2 | 18 | 51.30877193 | 0.002776394 | 0.071912931 |
| GO:0045893 | positive regulation of transcription, DNA-dependent | 6 | 491 | 5.642919927 | 0.002861106 | 0.071912931 |
| GO:0007173 | epidermal growth factor receptor signaling pathway | 4 | 185 | 9.984409673 | 0.002890391 | 0.071912931 |
| GO:0002040 | sprouting angiogenesis | 2 | 20 | 46.17789474 | 0.003438096 | 0.074831379 |
| GO:0046580 | negative regulation of Ras protein signal transduction | 2 | 21 | 43.97894737 | 0.003794646 | 0.074831379 |
| GO:0048565 | digestive tract development | 2 | 21 | 43.97894737 | 0.003794646 | 0.074831379 |
| GO:0001525 | angiogenesis | 4 | 201 | 9.189630793 | 0.003933352 | 0.074831379 |
| GO:0001701 | in utero embryonic development | 4 | 206 | 8.966581502 | 0.004307223 | 0.074831379 |
| GO:0070373 | negative regulation of ERK1 and ERK2 cascade | 2 | 23 | 40.15469108 | 0.004558772 | 0.074831379 |
| GO:0000187 | activation of MAPK activity | 3 | 95 | 14.58249307 | 0.004715078 | 0.074831379 |
| GO:0030177 | positive regulation of Wnt receptor signaling pathway | 2 | 24 | 38.48157895 | 0.0049662 | 0.074831379 |
| GO:0019221 | cytokine-mediated signaling pathway | 4 | 216 | 8.551461988 | 0.005128634 | 0.074831379 |
| GO:0043552 | positive regulation of phosphatidylinositol 3-kinase activity | 2 | 25 | 36.94231579 | 0.005390441 | 0.074831379 |
| GO:0006352 | DNA-dependent transcription, initiation | 2 | 26 | 35.52145749 | 0.005831422 | 0.074831379 |
| GO:0007049 | cell cycle | 4 | 224 | 8.246052632 | 0.005860167 | 0.074831379 |
| GO:0007528 | neuromuscular junction development | 2 | 28 | 32.98421053 | 0.006763309 | 0.074831379 |
| GO:0042127 | regulation of cell proliferation | 3 | 108 | 12.82719298 | 0.006814873 | 0.074831379 |
| GO:0007267 | cell-cell signaling | 4 | 242 | 7.632709874 | 0.00776656 | 0.074831379 |
| GO:0043407 | negative regulation of MAP kinase activity | 2 | 31 | 29.79219015 | 0.008284868 | 0.074831379 |
| GO:0002371 | dendritic cell cytokine production | 1 | 1 | 461.7789474 | 0.008662153 | 0.074831379 |
| GO:0003131 | mesodermal-endodermal cell signaling | 1 | 1 | 461.7789474 | 0.008662153 | 0.074831379 |
| GO:0010453 | regulation of cell fate commitment | 1 | 1 | 461.7789474 | 0.008662153 | 0.074831379 |
| GO:0021503 | neural fold bending | 1 | 1 | 461.7789474 | 0.008662153 | 0.074831379 |
| GO:0021769 | orbitofrontal cortex development | 1 | 1 | 461.7789474 | 0.008662153 | 0.074831379 |
| GO:0031052 | chromosome breakage | 1 | 1 | 461.7789474 | 0.008662153 | 0.074831379 |
| GO:0032762 | mast cell cytokine production | 1 | 1 | 461.7789474 | 0.008662153 | 0.074831379 |
| GO:0033091 | positive regulation of immature T cell proliferation | 1 | 1 | 461.7789474 | 0.008662153 | 0.074831379 |
| GO:0034721 | histone H3-K4 demethylation, trimethyl-H3-K4-specific | 1 | 1 | 461.7789474 | 0.008662153 | 0.074831379 |
| GO:0035602 | fibroblast growth factor receptor signaling pathway involved in negative regulation of apoptotic process in bone marrow | 1 | 1 | 461.7789474 | 0.008662153 | 0.074831379 |
| GO:0035603 | fibroblast growth factor receptor signaling pathway involved in hemopoiesis | 1 | 1 | 461.7789474 | 0.008662153 | 0.074831379 |
| GO:0035604 | fibroblast growth factor receptor signaling pathway involved in positive regulation of cell proliferation in bone marrow | 1 | 1 | 461.7789474 | 0.008662153 | 0.074831379 |
| GO:0035978 | histone H2A-S139 phosphorylation | 1 | 1 | 461.7789474 | 0.008662153 | 0.074831379 |
| GO:0042666 | negative regulation of ectodermal cell fate specification | 1 | 1 | 461.7789474 | 0.008662153 | 0.074831379 |
| GO:0046885 | regulation of hormone biosynthetic process | 1 | 1 | 461.7789474 | 0.008662153 | 0.074831379 |
| GO:0052565 | response to defense-related host nitric oxide production | 1 | 1 | 461.7789474 | 0.008662153 | 0.074831379 |
| GO:0060365 | coronal suture morphogenesis | 1 | 1 | 461.7789474 | 0.008662153 | 0.074831379 |
| GO:0060601 | lateral sprouting from an epithelium | 1 | 1 | 461.7789474 | 0.008662153 | 0.074831379 |
| GO:0061011 | hepatic duct development | 1 | 1 | 461.7789474 | 0.008662153 | 0.074831379 |
| GO:0070662 | mast cell proliferation | 1 | 1 | 461.7789474 | 0.008662153 | 0.074831379 |
| GO:0070673 | response to interleukin-18 | 1 | 1 | 461.7789474 | 0.008662153 | 0.074831379 |
| GO:0071103 | DNA conformation change | 1 | 1 | 461.7789474 | 0.008662153 | 0.074831379 |
| GO:0071228 | cellular response to tumor cell | 1 | 1 | 461.7789474 | 0.008662153 | 0.074831379 |
| GO:0097324 | melanocyte migration | 1 | 1 | 461.7789474 | 0.008662153 | 0.074831379 |
| GO:0097326 | melanocyte adhesion | 1 | 1 | 461.7789474 | 0.008662153 | 0.074831379 |
| GO:1900451 | positive regulation of glutamate receptor signaling pathway | 1 | 1 | 461.7789474 | 0.008662153 | 0.074831379 |
| GO:1900452 | regulation of long term synaptic depression | 1 | 1 | 461.7789474 | 0.008662153 | 0.074831379 |
| GO:2000351 | regulation of endothelial cell apoptotic process | 1 | 1 | 461.7789474 | 0.008662153 | 0.074831379 |
| GO:2000685 | positive regulation of cellular response to X-ray | 1 | 1 | 461.7789474 | 0.008662153 | 0.074831379 |
| GO:2000822 | regulation of behavioral fear response | 1 | 1 | 461.7789474 | 0.008662153 | 0.074831379 |
| GO:2001033 | negative regulation of double-strand break repair via nonhomologous end joining | 1 | 1 | 461.7789474 | 0.008662153 | 0.074831379 |
| GO:0035019 | somatic stem cell maintenance | 2 | 33 | 27.98660287 | 0.009380887 | 0.07885016 |
| GO:0042059 | negative regulation of epidermal growth factor receptor signaling pathway | 2 | 33 | 27.98660287 | 0.009380887 | 0.07885016 |
| GO:0030855 | epithelial cell differentiation | 2 | 36 | 25.65438596 | 0.011145955 | 0.09122181 |
| GO:0045892 | negative regulation of transcription, DNA-dependent | 5 | 446 | 5.176894029 | 0.011637407 | 0.09122181 |
| GO:0007155 | cell adhesion | 5 | 454 | 5.085671227 | 0.012553912 | 0.09122181 |
| GO:0001657 | ureteric bud development | 2 | 40 | 23.08894737 | 0.013722347 | 0.09122181 |
| GO:0051259 | protein oligomerization | 2 | 40 | 23.08894737 | 0.013722347 | 0.09122181 |
| GO:0035872 | nucleotide-binding domain, leucine rich repeat containing receptor signaling pathway | 2 | 42 | 21.98947368 | 0.015104837 | 0.09122181 |
| GO:0051301 | cell division | 4 | 295 | 6.261409456 | 0.015795169 | 0.09122181 |
| GO:0043066 | negative regulation of apoptotic process | 5 | 484 | 4.770443671 | 0.016458288 | 0.09122181 |
| GO:0001928 | regulation of exocyst assembly | 1 | 2 | 230.8894737 | 0.017305745 | 0.09122181 |
| GO:0002327 | immature B cell differentiation | 1 | 2 | 230.8894737 | 0.017305745 | 0.09122181 |
| GO:0002329 | pre-B cell differentiation | 1 | 2 | 230.8894737 | 0.017305745 | 0.09122181 |
| GO:0021773 | striatal medium spiny neuron differentiation | 1 | 2 | 230.8894737 | 0.017305745 | 0.09122181 |
| GO:0021849 | neuroblast division in subventricular zone | 1 | 2 | 230.8894737 | 0.017305745 | 0.09122181 |
| GO:0031507 | heterochromatin assembly | 1 | 2 | 230.8894737 | 0.017305745 | 0.09122181 |
| GO:0032803 | regulation of low-density lipoprotein particle receptor catabolic process | 1 | 2 | 230.8894737 | 0.017305745 | 0.09122181 |
| GO:0035607 | fibroblast growth factor receptor signaling pathway involved in orbitofrontal cortex development | 1 | 2 | 230.8894737 | 0.017305745 | 0.09122181 |
| GO:0038093 | Fc receptor signaling pathway | 1 | 2 | 230.8894737 | 0.017305745 | 0.09122181 |
| GO:0038109 | Kit signaling pathway | 1 | 2 | 230.8894737 | 0.017305745 | 0.09122181 |
| GO:0038162 | erythropoietin-mediated signaling pathway | 1 | 2 | 230.8894737 | 0.017305745 | 0.09122181 |
| GO:0042998 | positive regulation of Golgi to plasma membrane protein transport | 1 | 2 | 230.8894737 | 0.017305745 | 0.09122181 |
| GO:0045794 | negative regulation of cell volume | 1 | 2 | 230.8894737 | 0.017305745 | 0.09122181 |
| GO:0048333 | mesodermal cell differentiation | 1 | 2 | 230.8894737 | 0.017305745 | 0.09122181 |
| GO:0051036 | regulation of endosome size | 1 | 2 | 230.8894737 | 0.017305745 | 0.09122181 |
| GO:0060123 | regulation of growth hormone secretion | 1 | 2 | 230.8894737 | 0.017305745 | 0.09122181 |
| GO:0060431 | primary lung bud formation | 1 | 2 | 230.8894737 | 0.017305745 | 0.09122181 |
| GO:0060437 | lung growth | 1 | 2 | 230.8894737 | 0.017305745 | 0.09122181 |
| GO:0060512 | prostate gland morphogenesis | 1 | 2 | 230.8894737 | 0.017305745 | 0.09122181 |
| GO:0060529 | squamous basal epithelial stem cell differentiation involved in prostate gland acinus development | 1 | 2 | 230.8894737 | 0.017305745 | 0.09122181 |
| GO:0060595 | fibroblast growth factor receptor signaling pathway involved in mammary gland specification | 1 | 2 | 230.8894737 | 0.017305745 | 0.09122181 |
| GO:0060615 | mammary gland bud formation | 1 | 2 | 230.8894737 | 0.017305745 | 0.09122181 |
| GO:0060667 | branch elongation involved in salivary gland morphogenesis | 1 | 2 | 230.8894737 | 0.017305745 | 0.09122181 |
| GO:0060915 | mesenchymal cell differentiation involved in lung development | 1 | 2 | 230.8894737 | 0.017305745 | 0.09122181 |
| GO:0061017 | hepatoblast differentiation | 1 | 2 | 230.8894737 | 0.017305745 | 0.09122181 |
| GO:0070427 | nucleotide-binding oligomerization domain containing 1 signaling pathway | 1 | 2 | 230.8894737 | 0.017305745 | 0.09122181 |
| GO:0070671 | response to interleukin-12 | 1 | 2 | 230.8894737 | 0.017305745 | 0.09122181 |
| GO:0072014 | proximal tubule development | 1 | 2 | 230.8894737 | 0.017305745 | 0.09122181 |
| GO:0090402 | oncogene-induced senescence | 1 | 2 | 230.8894737 | 0.017305745 | 0.09122181 |
| GO:0097107 | postsynaptic density assembly | 1 | 2 | 230.8894737 | 0.017305745 | 0.09122181 |
| GO:0097117 | guanylate kinase-associated protein clustering | 1 | 2 | 230.8894737 | 0.017305745 | 0.09122181 |
| GO:0097118 | neuroligin clustering | 1 | 2 | 230.8894737 | 0.017305745 | 0.09122181 |
| GO:1900271 | regulation of long-term synaptic potentiation | 1 | 2 | 230.8894737 | 0.017305745 | 0.09122181 |
| GO:2000824 | negative regulation of androgen receptor activity | 1 | 2 | 230.8894737 | 0.017305745 | 0.09122181 |
| GO:2001038 | regulation of cellular response to drug | 1 | 2 | 230.8894737 | 0.017305745 | 0.09122181 |
| GO:2001137 | positive regulation of endocytic recycling | 1 | 2 | 230.8894737 | 0.017305745 | 0.09122181 |
| GO:0014068 | positive regulation of phosphatidylinositol 3-kinase cascade | 2 | 46 | 20.07734554 | 0.018055632 | 0.092814901 |
| GO:0019827 | stem cell maintenance | 2 | 46 | 20.07734554 | 0.018055632 | 0.092814901 |
| GO:0051781 | positive regulation of cell division | 2 | 46 | 20.07734554 | 0.018055632 | 0.092814901 |
| GO:0045665 | negative regulation of neuron differentiation | 2 | 48 | 19.24078947 | 0.019622835 | 0.100044292 |
| GO:0033138 | positive regulation of peptidyl-serine phosphorylation | 2 | 51 | 18.10897833 | 0.022086865 | 0.102732275 |
| GO:0001938 | positive regulation of endothelial cell proliferation | 2 | 52 | 17.76072874 | 0.022938131 | 0.102732275 |
| GO:0016525 | negative regulation of angiogenesis | 2 | 53 | 17.42562066 | 0.023804246 | 0.102732275 |
| GO:0046330 | positive regulation of JNK cascade | 2 | 53 | 17.42562066 | 0.023804246 | 0.102732275 |
| GO:0001838 | embryonic epithelial tube formation | 1 | 3 | 153.9263158 | 0.025930815 | 0.102732275 |
| GO:0002573 | myeloid leukocyte differentiation | 1 | 3 | 153.9263158 | 0.025930815 | 0.102732275 |
| GO:0003199 | endocardial cushion to mesenchymal transition involved in heart valve formation | 1 | 3 | 153.9263158 | 0.025930815 | 0.102732275 |
| GO:0010621 | negative regulation of transcription by transcription factor localization | 1 | 3 | 153.9263158 | 0.025930815 | 0.102732275 |
| GO:0010883 | regulation of lipid storage | 1 | 3 | 153.9263158 | 0.025930815 | 0.102732275 |
| GO:0014028 | notochord formation | 1 | 3 | 153.9263158 | 0.025930815 | 0.102732275 |
| GO:0014834 | satellite cell maintenance involved in skeletal muscle regeneration | 1 | 3 | 153.9263158 | 0.025930815 | 0.102732275 |
| GO:0016576 | histone dephosphorylation | 1 | 3 | 153.9263158 | 0.025930815 | 0.102732275 |
| GO:0021800 | cerebral cortex tangential migration | 1 | 3 | 153.9263158 | 0.025930815 | 0.102732275 |
| GO:0021847 | ventricular zone neuroblast division | 1 | 3 | 153.9263158 | 0.025930815 | 0.102732275 |
| GO:0032232 | negative regulation of actin filament bundle assembly | 1 | 3 | 153.9263158 | 0.025930815 | 0.102732275 |
| GO:0032313 | regulation of Rab GTPase activity | 1 | 3 | 153.9263158 | 0.025930815 | 0.102732275 |
| GO:0032878 | regulation of establishment or maintenance of cell polarity | 1 | 3 | 153.9263158 | 0.025930815 | 0.102732275 |
| GO:0033688 | regulation of osteoblast proliferation | 1 | 3 | 153.9263158 | 0.025930815 | 0.102732275 |
| GO:0035022 | positive regulation of Rac protein signal transduction | 1 | 3 | 153.9263158 | 0.025930815 | 0.102732275 |
| GO:0035986 | senescence-associated heterochromatin focus assembly | 1 | 3 | 153.9263158 | 0.025930815 | 0.102732275 |
| GO:0035987 | endodermal cell differentiation | 1 | 3 | 153.9263158 | 0.025930815 | 0.102732275 |
| GO:0048014 | Tie signaling pathway | 1 | 3 | 153.9263158 | 0.025930815 | 0.102732275 |
| GO:0060374 | mast cell differentiation | 1 | 3 | 153.9263158 | 0.025930815 | 0.102732275 |
| GO:0060449 | bud elongation involved in lung branching | 1 | 3 | 153.9263158 | 0.025930815 | 0.102732275 |
| GO:0060523 | prostate epithelial cord elongation | 1 | 3 | 153.9263158 | 0.025930815 | 0.102732275 |
| GO:0060916 | mesenchymal cell proliferation involved in lung development | 1 | 3 | 153.9263158 | 0.025930815 | 0.102732275 |
| GO:0060964 | regulation of gene silencing by miRNA | 1 | 3 | 153.9263158 | 0.025930815 | 0.102732275 |
| GO:0061009 | common bile duct development | 1 | 3 | 153.9263158 | 0.025930815 | 0.102732275 |
| GO:0061010 | gall bladder development | 1 | 3 | 153.9263158 | 0.025930815 | 0.102732275 |
| GO:0071224 | cellular response to peptidoglycan | 1 | 3 | 153.9263158 | 0.025930815 | 0.102732275 |
| GO:0097113 | alpha-amino-3-hydroxy-5-methyl-4-isoxazole propionate receptor clustering | 1 | 3 | 153.9263158 | 0.025930815 | 0.102732275 |
| GO:0097116 | gephyrin clustering | 1 | 3 | 153.9263158 | 0.025930815 | 0.102732275 |
| GO:1901741 | positive regulation of myoblast fusion | 1 | 3 | 153.9263158 | 0.025930815 | 0.102732275 |
| GO:2000821 | regulation of grooming behavior | 1 | 3 | 153.9263158 | 0.025930815 | 0.102732275 |
| GO:2000969 | positive regulation of alpha-amino-3-hydroxy-5-methyl-4-isoxazole propionate selective glutamate receptor activity | 1 | 3 | 153.9263158 | 0.025930815 | 0.102732275 |
| GO:0035264 | multicellular organism growth | 2 | 56 | 16.49210526 | 0.026491019 | 0.104287429 |
| GO:0042472 | inner ear morphogenesis | 2 | 57 | 16.20277008 | 0.027415864 | 0.107249482 |
| GO:0043410 | positive regulation of MAPK cascade | 2 | 64 | 14.43059211 | 0.034292581 | 0.116751439 |
| GO:0000395 | mRNA 5'-splice site recognition | 1 | 4 | 115.4447368 | 0.034537403 | 0.116751439 |
| GO:0003183 | mitral valve morphogenesis | 1 | 4 | 115.4447368 | 0.034537403 | 0.116751439 |
| GO:0010586 | miRNA metabolic process | 1 | 4 | 115.4447368 | 0.034537403 | 0.116751439 |
| GO:0010989 | negative regulation of low-density lipoprotein particle clearance | 1 | 4 | 115.4447368 | 0.034537403 | 0.116751439 |
| GO:0030091 | protein repair | 1 | 4 | 115.4447368 | 0.034537403 | 0.116751439 |
| GO:0031503 | protein complex localization | 1 | 4 | 115.4447368 | 0.034537403 | 0.116751439 |
| GO:0032808 | lacrimal gland development | 1 | 4 | 115.4447368 | 0.034537403 | 0.116751439 |
| GO:0035234 | germ cell programmed cell death | 1 | 4 | 115.4447368 | 0.034537403 | 0.116751439 |
| GO:0035412 | regulation of catenin import into nucleus | 1 | 4 | 115.4447368 | 0.034537403 | 0.116751439 |
| GO:0038031 | non-canonical Wnt receptor signaling pathway via JNK cascade | 1 | 4 | 115.4447368 | 0.034537403 | 0.116751439 |
| GO:0043535 | regulation of blood vessel endothelial cell migration | 1 | 4 | 115.4447368 | 0.034537403 | 0.116751439 |
| GO:0048070 | regulation of developmental pigmentation | 1 | 4 | 115.4447368 | 0.034537403 | 0.116751439 |
| GO:0048755 | branching morphogenesis of a nerve | 1 | 4 | 115.4447368 | 0.034537403 | 0.116751439 |
| GO:0051782 | negative regulation of cell division | 1 | 4 | 115.4447368 | 0.034537403 | 0.116751439 |
| GO:0051835 | positive regulation of synapse structural plasticity | 1 | 4 | 115.4447368 | 0.034537403 | 0.116751439 |
| GO:0060613 | fat pad development | 1 | 4 | 115.4447368 | 0.034537403 | 0.116751439 |
| GO:0061002 | negative regulation of dendritic spine morphogenesis | 1 | 4 | 115.4447368 | 0.034537403 | 0.116751439 |
| GO:0061031 | endodermal digestive tract morphogenesis | 1 | 4 | 115.4447368 | 0.034537403 | 0.116751439 |
| GO:0065004 | protein-DNA complex assembly | 1 | 4 | 115.4447368 | 0.034537403 | 0.116751439 |
| GO:0070431 | nucleotide-binding oligomerization domain containing 2 signaling pathway | 1 | 4 | 115.4447368 | 0.034537403 | 0.116751439 |
| GO:0097114 | N-methyl-D-aspartate receptor clustering | 1 | 4 | 115.4447368 | 0.034537403 | 0.116751439 |
| GO:2000177 | regulation of neural precursor cell proliferation | 1 | 4 | 115.4447368 | 0.034537403 | 0.116751439 |
| GO:2000637 | positive regulation of gene silencing by miRNA | 1 | 4 | 115.4447368 | 0.034537403 | 0.116751439 |
| GO:2000774 | positive regulation of cellular senescence | 1 | 4 | 115.4447368 | 0.034537403 | 0.116751439 |
| GO:0000165 | MAPK cascade | 2 | 66 | 13.99330144 | 0.036384874 | 0.121997614 |
| GO:0043065 | positive regulation of apoptotic process | 3 | 197 | 7.032166711 | 0.036481602 | 0.121997614 |
| GO:0051897 | positive regulation of protein kinase B signaling cascade | 2 | 68 | 13.58173375 | 0.038532894 | 0.128101831 |
| GO:0030154 | cell differentiation | 4 | 382 | 4.835381648 | 0.038718881 | 0.128101831 |
| GO:0008584 | male gonad development | 2 | 72 | 12.82719298 | 0.042994078 | 0.12834493 |
| GO:0002318 | myeloid progenitor cell differentiation | 1 | 5 | 92.35578947 | 0.043125547 | 0.12834493 |
| GO:0002551 | mast cell chemotaxis | 1 | 5 | 92.35578947 | 0.043125547 | 0.12834493 |
| GO:0003149 | membranous septum morphogenesis | 1 | 5 | 92.35578947 | 0.043125547 | 0.12834493 |
| GO:0003180 | aortic valve morphogenesis | 1 | 5 | 92.35578947 | 0.043125547 | 0.12834493 |
| GO:0010543 | regulation of platelet activation | 1 | 5 | 92.35578947 | 0.043125547 | 0.12834493 |
| GO:0014031 | mesenchymal cell development | 1 | 5 | 92.35578947 | 0.043125547 | 0.12834493 |
| GO:0021853 | cerebral cortex GABAergic interneuron migration | 1 | 5 | 92.35578947 | 0.043125547 | 0.12834493 |
| GO:0022612 | gland morphogenesis | 1 | 5 | 92.35578947 | 0.043125547 | 0.12834493 |
| GO:0030853 | negative regulation of granulocyte differentiation | 1 | 5 | 92.35578947 | 0.043125547 | 0.12834493 |
| GO:0034720 | histone H3-K4 demethylation | 1 | 5 | 92.35578947 | 0.043125547 | 0.12834493 |
| GO:0035278 | negative regulation of translation involved in gene silencing by miRNA | 1 | 5 | 92.35578947 | 0.043125547 | 0.12834493 |
| GO:0035988 | chondrocyte proliferation | 1 | 5 | 92.35578947 | 0.043125547 | 0.12834493 |
| GO:0043457 | regulation of cellular respiration | 1 | 5 | 92.35578947 | 0.043125547 | 0.12834493 |
| GO:0045627 | positive regulation of T-helper 1 cell differentiation | 1 | 5 | 92.35578947 | 0.043125547 | 0.12834493 |
| GO:0048170 | positive regulation of long-term neuronal synaptic plasticity | 1 | 5 | 92.35578947 | 0.043125547 | 0.12834493 |
| GO:0048489 | synaptic vesicle transport | 1 | 5 | 92.35578947 | 0.043125547 | 0.12834493 |
| GO:0048608 | reproductive structure development | 1 | 5 | 92.35578947 | 0.043125547 | 0.12834493 |
| GO:0060054 | positive regulation of epithelial cell proliferation involved in wound healing | 1 | 5 | 92.35578947 | 0.043125547 | 0.12834493 |
| GO:0097104 | postsynaptic membrane assembly | 1 | 5 | 92.35578947 | 0.043125547 | 0.12834493 |
| GO:0097119 | postsynaptic density protein 95 clustering | 1 | 5 | 92.35578947 | 0.043125547 | 0.12834493 |
| GO:0007229 | integrin-mediated signaling pathway | 2 | 74 | 12.48051209 | 0.045306229 | 0.134192735 |
| **Significant GO affected by dwon-regulated miRNAs targets** | | | | | | |
| GO:0007165 | signal transduction | 19 | 1030 | 5.950249857 | 2.14231E-09 | 1.70314E-06 |
| GO:0007264 | small GTPase mediated signal transduction | 11 | 336 | 10.56020221 | 3.75702E-08 | 1.49341E-05 |
| GO:0006897 | endocytosis | 6 | 115 | 16.82953964 | 6.77691E-06 | 0.001795881 |
| GO:0000122 | negative regulation of transcription from RNA polymerase II promoter | 10 | 509 | 6.337252976 | 1.86885E-05 | 0.003714339 |
| GO:0006915 | apoptotic process | 11 | 654 | 5.425424987 | 2.69269E-05 | 0.004281373 |
| GO:0032877 | positive regulation of DNA endoreduplication | 2 | 2 | 322.5661765 | 3.81616E-05 | 0.005056418 |
| GO:0010881 | regulation of cardiac muscle contraction by regulation of the release of sequestered calcium ion | 3 | 15 | 64.51323529 | 5.16119E-05 | 0.005596838 |
| GO:0045944 | positive regulation of transcription from RNA polymerase II promoter | 11 | 708 | 5.011621386 | 5.63204E-05 | 0.005596838 |
| GO:0032466 | negative regulation of cytokinesis | 2 | 3 | 215.0441176 | 0.000114252 | 0.009240244 |
| GO:0007399 | nervous system development | 7 | 283 | 7.978668676 | 0.000124019 | 0.009240244 |
| GO:0002040 | sprouting angiogenesis | 3 | 20 | 48.38492647 | 0.000127852 | 0.009240244 |
| GO:0055085 | transmembrane transport | 9 | 538 | 5.396088454 | 0.000198269 | 0.01313529 |
| GO:0060718 | chorionic trophoblast cell differentiation | 2 | 4 | 161.2830882 | 0.000228038 | 0.013945423 |
| GO:0006468 | protein phosphorylation | 7 | 338 | 6.680364601 | 0.000378113 | 0.020102387 |
| GO:0055013 | cardiac muscle cell development | 2 | 5 | 129.0264706 | 0.00037929 | 0.020102387 |
| GO:0007154 | cell communication | 4 | 81 | 15.9291939 | 0.000488716 | 0.023944025 |
| GO:0001666 | response to hypoxia | 5 | 155 | 10.40536053 | 0.000512011 | 0.023944025 |
| GO:0010763 | positive regulation of fibroblast migration | 2 | 7 | 92.16176471 | 0.000793272 | 0.033192152 |
| GO:0055119 | relaxation of cardiac muscle | 2 | 7 | 92.16176471 | 0.000793272 | 0.033192152 |
| GO:0042493 | response to drug | 6 | 274 | 7.063492915 | 0.000896474 | 0.035634839 |
| GO:0046325 | negative regulation of glucose import | 2 | 8 | 80.64154412 | 0.001055544 | 0.038143522 |
| GO:0070365 | hepatocyte differentiation | 2 | 8 | 80.64154412 | 0.001055544 | 0.038143522 |
| GO:0002544 | chronic inflammatory response | 2 | 9 | 71.68137255 | 0.001354368 | 0.044863448 |
| GO:0010832 | negative regulation of myotube differentiation | 2 | 9 | 71.68137255 | 0.001354368 | 0.044863448 |
| GO:0010613 | positive regulation of cardiac muscle hypertrophy | 2 | 10 | 64.51323529 | 0.001689518 | 0.047970255 |
| GO:0060314 | regulation of ryanodine-sensitive calcium-release channel activity | 2 | 10 | 64.51323529 | 0.001689518 | 0.047970255 |
| GO:0060402 | calcium ion transport into cytosol | 2 | 10 | 64.51323529 | 0.001689518 | 0.047970255 |
| GO:0060707 | trophoblast giant cell differentiation | 2 | 10 | 64.51323529 | 0.001689518 | 0.047970255 |
| GO:0045892 | negative regulation of transcription, DNA-dependent | 7 | 446 | 5.062697837 | 0.002029888 | 0.055646932 |
| GO:0033138 | positive regulation of peptidyl-serine phosphorylation | 3 | 51 | 18.97448097 | 0.00217707 | 0.057692348 |
| GO:0043547 | positive regulation of GTPase activity | 3 | 54 | 17.92034314 | 0.002575507 | 0.066049292 |
| GO:0051044 | positive regulation of membrane protein ectodomain proteolysis | 2 | 13 | 49.62556561 | 0.002910682 | 0.072312254 |
| GO:0043066 | negative regulation of apoptotic process | 7 | 484 | 4.665213296 | 0.003278316 | 0.076976405 |
| GO:0002028 | regulation of sodium ion transport | 2 | 14 | 46.08088235 | 0.003388898 | 0.076976405 |
| GO:0071044 | histone mRNA catabolic process | 2 | 14 | 46.08088235 | 0.003388898 | 0.076976405 |
| GO:0016044 | cellular membrane organization | 4 | 143 | 9.022830111 | 0.004230818 | 0.093430573 |
| GO:0015031 | protein transport | 6 | 388 | 4.98813675 | 0.005517284 | 0.106004755 |
| GO:0048011 | neurotrophin TRK receptor signaling pathway | 5 | 269 | 5.995653838 | 0.006259032 | 0.106004755 |
| GO:0006351 | transcription, DNA-dependent | 14 | 1827 | 2.471771467 | 0.006671514 | 0.106004755 |
| GO:0051056 | regulation of small GTPase mediated signal transduction | 4 | 163 | 7.915734392 | 0.006845955 | 0.106004755 |
| GO:0001932 | regulation of protein phosphorylation | 2 | 20 | 32.25661765 | 0.006990001 | 0.106004755 |
| GO:0006811 | ion transport | 4 | 165 | 7.819786096 | 0.007157335 | 0.106004755 |
| GO:0008284 | positive regulation of cell proliferation | 6 | 411 | 4.708995277 | 0.007374298 | 0.106004755 |
| GO:0030316 | osteoclast differentiation | 2 | 21 | 30.72058824 | 0.007710125 | 0.106004755 |
| GO:0034605 | cellular response to heat | 2 | 21 | 30.72058824 | 0.007710125 | 0.106004755 |
| GO:0009791 | post-embryonic development | 3 | 80 | 12.09623162 | 0.00804486 | 0.106004755 |
| GO:0002576 | platelet degranulation | 3 | 81 | 11.94689542 | 0.008335481 | 0.106004755 |
| GO:0002027 | regulation of heart rate | 2 | 22 | 29.32419786 | 0.008463944 | 0.106004755 |
| GO:0006954 | inflammatory response | 5 | 295 | 5.46722333 | 0.009345606 | 0.106004755 |
| GO:0030177 | positive regulation of Wnt receptor signaling pathway | 2 | 24 | 26.88051471 | 0.010071819 | 0.106004755 |
| GO:0007517 | muscle organ development | 3 | 88 | 10.9965742 | 0.010553224 | 0.106004755 |
| GO:0031572 | G2 DNA damage checkpoint | 2 | 25 | 25.80529412 | 0.010925453 | 0.106004755 |
| GO:0060079 | regulation of excitatory postsynaptic membrane potential | 2 | 26 | 24.81278281 | 0.011811937 | 0.106004755 |
| GO:0000921 | septin ring assembly | 1 | 1 | 322.5661765 | 0.012400556 | 0.106004755 |
| GO:0002037 | negative regulation of L-glutamate transport | 1 | 1 | 322.5661765 | 0.012400556 | 0.106004755 |
| GO:0002581 | negative regulation of antigen processing and presentation of peptide or polysaccharide antigen via MHC class II | 1 | 1 | 322.5661765 | 0.012400556 | 0.106004755 |
| GO:0002605 | negative regulation of dendritic cell antigen processing and presentation | 1 | 1 | 322.5661765 | 0.012400556 | 0.106004755 |
| GO:0003017 | lymph circulation | 1 | 1 | 322.5661765 | 0.012400556 | 0.106004755 |
| GO:0010635 | regulation of mitochondrial fusion | 1 | 1 | 322.5661765 | 0.012400556 | 0.106004755 |
| GO:0010751 | negative regulation of nitric oxide mediated signal transduction | 1 | 1 | 322.5661765 | 0.012400556 | 0.106004755 |
| GO:0010754 | negative regulation of cGMP-mediated signaling | 1 | 1 | 322.5661765 | 0.012400556 | 0.106004755 |
| GO:0014076 | response to fluoxetine | 1 | 1 | 322.5661765 | 0.012400556 | 0.106004755 |
| GO:0014820 | tonic smooth muscle contraction | 1 | 1 | 322.5661765 | 0.012400556 | 0.106004755 |
| GO:0015675 | nickel cation transport | 1 | 1 | 322.5661765 | 0.012400556 | 0.106004755 |
| GO:0015676 | vanadium ion transport | 1 | 1 | 322.5661765 | 0.012400556 | 0.106004755 |
| GO:0015684 | ferrous iron transport | 1 | 1 | 322.5661765 | 0.012400556 | 0.106004755 |
| GO:0015692 | lead ion transport | 1 | 1 | 322.5661765 | 0.012400556 | 0.106004755 |
| GO:0015734 | taurine transport | 1 | 1 | 322.5661765 | 0.012400556 | 0.106004755 |
| GO:0030801 | positive regulation of cyclic nucleotide metabolic process | 1 | 1 | 322.5661765 | 0.012400556 | 0.106004755 |
| GO:0032509 | endosome transport via multivesicular body sorting pathway | 1 | 1 | 322.5661765 | 0.012400556 | 0.106004755 |
| GO:0033301 | cell cycle comprising mitosis without cytokinesis | 1 | 1 | 322.5661765 | 0.012400556 | 0.106004755 |
| GO:0035444 | nickel cation transmembrane transport | 1 | 1 | 322.5661765 | 0.012400556 | 0.106004755 |
| GO:0035645 | enteric smooth muscle cell differentiation | 1 | 1 | 322.5661765 | 0.012400556 | 0.106004755 |
| GO:0038089 | positive regulation of cell migration by vascular endothelial growth factor signaling pathway | 1 | 1 | 322.5661765 | 0.012400556 | 0.106004755 |
| GO:0044557 | relaxation of smooth muscle | 1 | 1 | 322.5661765 | 0.012400556 | 0.106004755 |
| GO:0045368 | positive regulation of interleukin-13 biosynthetic process | 1 | 1 | 322.5661765 | 0.012400556 | 0.106004755 |
| GO:0045622 | regulation of T-helper cell differentiation | 1 | 1 | 322.5661765 | 0.012400556 | 0.106004755 |
| GO:0045994 | positive regulation of translational initiation by iron | 1 | 1 | 322.5661765 | 0.012400556 | 0.106004755 |
| GO:0051121 | hepoxilin metabolic process | 1 | 1 | 322.5661765 | 0.012400556 | 0.106004755 |
| GO:0060453 | regulation of gastric acid secretion | 1 | 1 | 322.5661765 | 0.012400556 | 0.106004755 |
| GO:0061048 | negative regulation of branching involved in lung morphogenesis | 1 | 1 | 322.5661765 | 0.012400556 | 0.106004755 |
| GO:0070346 | positive regulation of fat cell proliferation | 1 | 1 | 322.5661765 | 0.012400556 | 0.106004755 |
| GO:0070627 | ferrous iron import | 1 | 1 | 322.5661765 | 0.012400556 | 0.106004755 |
| GO:0071677 | positive regulation of mononuclear cell migration | 1 | 1 | 322.5661765 | 0.012400556 | 0.106004755 |
| GO:0072105 | ureteric peristalsis | 1 | 1 | 322.5661765 | 0.012400556 | 0.106004755 |
| GO:0072195 | kidney smooth muscle cell differentiation | 1 | 1 | 322.5661765 | 0.012400556 | 0.106004755 |
| GO:0072347 | response to anesthetic | 1 | 1 | 322.5661765 | 0.012400556 | 0.106004755 |
| GO:0085032 | modulation by symbiont of host I-kappaB kinase/NF-kappaB cascade | 1 | 1 | 322.5661765 | 0.012400556 | 0.106004755 |
| GO:0086100 | endothelin receptor signaling pathway | 1 | 1 | 322.5661765 | 0.012400556 | 0.106004755 |
| GO:0090298 | negative regulation of mitochondrial DNA replication | 1 | 1 | 322.5661765 | 0.012400556 | 0.106004755 |
| GO:0097369 | sodium ion import | 1 | 1 | 322.5661765 | 0.012400556 | 0.106004755 |
| GO:1900748 | positive regulation of vascular endothelial growth factor signaling pathway | 1 | 1 | 322.5661765 | 0.012400556 | 0.106004755 |
| GO:1901660 | calcium ion export | 1 | 1 | 322.5661765 | 0.012400556 | 0.106004755 |
| GO:0043507 | positive regulation of JUN kinase activity | 2 | 27 | 23.89379085 | 0.012731062 | 0.106538886 |
| GO:2000379 | positive regulation of reactive oxygen species metabolic process | 2 | 27 | 23.89379085 | 0.012731062 | 0.106538886 |
| GO:0000187 | activation of MAPK activity | 3 | 95 | 10.18630031 | 0.013103347 | 0.107393408 |
| GO:0006936 | muscle contraction | 3 | 95 | 10.18630031 | 0.013103347 | 0.107393408 |
| GO:0002474 | antigen processing and presentation of peptide antigen via MHC class I | 3 | 97 | 9.976273499 | 0.013895118 | 0.112720602 |
| GO:0097190 | apoptotic signaling pathway | 3 | 100 | 9.676985294 | 0.01513665 | 0.121551887 |
| GO:0010595 | positive regulation of endothelial cell migration | 2 | 31 | 20.81072106 | 0.016729836 | 0.131243639 |
| GO:0045071 | negative regulation of viral genome replication | 2 | 31 | 20.81072106 | 0.016729836 | 0.131243639 |
| GO:0006006 | glucose metabolic process | 3 | 105 | 9.216176471 | 0.017352121 | 0.131243639 |
| GO:0007219 | Notch signaling pathway | 3 | 108 | 8.960171569 | 0.01877078 | 0.131243639 |
| GO:0006909 | phagocytosis | 2 | 33 | 19.54946524 | 0.018919712 | 0.131243639 |
| GO:0045668 | negative regulation of osteoblast differentiation | 2 | 33 | 19.54946524 | 0.018919712 | 0.131243639 |
| GO:0006810 | transport | 5 | 350 | 4.608088235 | 0.019309126 | 0.131243639 |
| GO:0007611 | learning or memory | 2 | 34 | 18.97448097 | 0.020061562 | 0.131243639 |
| GO:0051865 | protein autoubiquitination | 2 | 34 | 18.97448097 | 0.020061562 | 0.131243639 |
| GO:0001890 | placenta development | 2 | 35 | 18.43235294 | 0.021234416 | 0.131243639 |
| GO:0060048 | cardiac muscle contraction | 2 | 35 | 18.43235294 | 0.021234416 | 0.131243639 |
| GO:0006184 | GTP catabolic process | 3 | 113 | 8.5637038 | 0.021287185 | 0.131243639 |
| GO:0007049 | cell cycle | 4 | 224 | 5.760110294 | 0.021344583 | 0.131243639 |
| GO:0030335 | positive regulation of cell migration | 3 | 114 | 8.488583591 | 0.021813558 | 0.131243639 |
| GO:0006605 | protein targeting | 2 | 37 | 17.43600954 | 0.02367234 | 0.131243639 |
| GO:0034220 | ion transmembrane transport | 3 | 118 | 8.200834995 | 0.023997062 | 0.131243639 |
| GO:0002876 | positive regulation of chronic inflammatory response to antigenic stimulus | 1 | 2 | 161.2830882 | 0.024762951 | 0.131243639 |
| GO:0003162 | atrioventricular node development | 1 | 2 | 161.2830882 | 0.024762951 | 0.131243639 |
| GO:0007497 | posterior midgut development | 1 | 2 | 161.2830882 | 0.024762951 | 0.131243639 |
| GO:0010882 | regulation of cardiac muscle contraction by calcium ion signaling | 1 | 2 | 161.2830882 | 0.024762951 | 0.131243639 |
| GO:0014043 | negative regulation of neuron maturation | 1 | 2 | 161.2830882 | 0.024762951 | 0.131243639 |
| GO:0021747 | cochlear nucleus development | 1 | 2 | 161.2830882 | 0.024762951 | 0.131243639 |
| GO:0031346 | positive regulation of cell projection organization | 1 | 2 | 161.2830882 | 0.024762951 | 0.131243639 |
| GO:0035434 | copper ion transmembrane transport | 1 | 2 | 161.2830882 | 0.024762951 | 0.131243639 |
| GO:0038180 | nerve growth factor signaling pathway | 1 | 2 | 161.2830882 | 0.024762951 | 0.131243639 |
| GO:0043314 | negative regulation of neutrophil degranulation | 1 | 2 | 161.2830882 | 0.024762951 | 0.131243639 |
| GO:0043652 | engulfment of apoptotic cell | 1 | 2 | 161.2830882 | 0.024762951 | 0.131243639 |
| GO:0045082 | positive regulation of interleukin-10 biosynthetic process | 1 | 2 | 161.2830882 | 0.024762951 | 0.131243639 |
| GO:0045608 | negative regulation of auditory receptor cell differentiation | 1 | 2 | 161.2830882 | 0.024762951 | 0.131243639 |
| GO:0048227 | plasma membrane to endosome transport | 1 | 2 | 161.2830882 | 0.024762951 | 0.131243639 |
| GO:0050779 | RNA destabilization | 1 | 2 | 161.2830882 | 0.024762951 | 0.131243639 |
| GO:0050975 | sensory perception of touch | 1 | 2 | 161.2830882 | 0.024762951 | 0.131243639 |
| GO:0051343 | positive regulation of cyclic-nucleotide phosphodiesterase activity | 1 | 2 | 161.2830882 | 0.024762951 | 0.131243639 |
| GO:0060282 | positive regulation of oocyte development | 1 | 2 | 161.2830882 | 0.024762951 | 0.131243639 |
| GO:0060341 | regulation of cellular localization | 1 | 2 | 161.2830882 | 0.024762951 | 0.131243639 |
| GO:0060401 | cytosolic calcium ion transport | 1 | 2 | 161.2830882 | 0.024762951 | 0.131243639 |
| GO:0060414 | aorta smooth muscle tissue morphogenesis | 1 | 2 | 161.2830882 | 0.024762951 | 0.131243639 |
| GO:0060557 | positive regulation of vitamin D biosynthetic process | 1 | 2 | 161.2830882 | 0.024762951 | 0.131243639 |
| GO:0060693 | regulation of branching involved in salivary gland morphogenesis | 1 | 2 | 161.2830882 | 0.024762951 | 0.131243639 |
| GO:0060928 | atrioventricular node cell development | 1 | 2 | 161.2830882 | 0.024762951 | 0.131243639 |
| GO:0061097 | regulation of protein tyrosine kinase activity | 1 | 2 | 161.2830882 | 0.024762951 | 0.131243639 |
| GO:0071396 | cellular response to lipid | 1 | 2 | 161.2830882 | 0.024762951 | 0.131243639 |
| GO:0071421 | manganese ion transmembrane transport | 1 | 2 | 161.2830882 | 0.024762951 | 0.131243639 |
| GO:0071436 | sodium ion export | 1 | 2 | 161.2830882 | 0.024762951 | 0.131243639 |
| GO:0072014 | proximal tubule development | 1 | 2 | 161.2830882 | 0.024762951 | 0.131243639 |
| GO:0072070 | loop of Henle development | 1 | 2 | 161.2830882 | 0.024762951 | 0.131243639 |
| GO:0072540 | T-helper 17 cell lineage commitment | 1 | 2 | 161.2830882 | 0.024762951 | 0.131243639 |
| GO:1901844 | regulation of cell communication by electrical coupling involved in cardiac conduction | 1 | 2 | 161.2830882 | 0.024762951 | 0.131243639 |
| GO:2000373 | positive regulation of DNA topoisomerase (ATP-hydrolyzing) activity | 1 | 2 | 161.2830882 | 0.024762951 | 0.131243639 |
| GO:2001170 | negative regulation of ATP biosynthetic process | 1 | 2 | 161.2830882 | 0.024762951 | 0.131243639 |
| GO:2001171 | positive regulation of ATP biosynthetic process | 1 | 2 | 161.2830882 | 0.024762951 | 0.131243639 |
| GO:0001755 | neural crest cell migration | 2 | 38 | 16.97716718 | 0.024937011 | 0.131290887 |
| GO:0006865 | amino acid transport | 2 | 39 | 16.5418552 | 0.026231892 | 0.13630297 |
| GO:0038032 | termination of G-protein coupled receptor signaling pathway | 2 | 39 | 16.5418552 | 0.026231892 | 0.13630297 |
| GO:0001657 | ureteric bud development | 2 | 40 | 16.12830882 | 0.027556786 | 0.141339646 |
| GO:0048839 | inner ear development | 2 | 40 | 16.12830882 | 0.027556786 | 0.141339646 |
| GO:0007610 | behavior | 2 | 41 | 15.73493544 | 0.028911497 | 0.146398983 |
| GO:0048661 | positive regulation of smooth muscle cell proliferation | 2 | 41 | 15.73493544 | 0.028911497 | 0.146398983 |
| GO:0014070 | response to organic cyclic compound | 2 | 43 | 15.00307798 | 0.031709593 | 0.149667024 |
| GO:0043085 | positive regulation of catalytic activity | 2 | 43 | 15.00307798 | 0.031709593 | 0.149667024 |
| GO:0010628 | positive regulation of gene expression | 3 | 131 | 7.387011675 | 0.031974235 | 0.149667024 |
| GO:0006520 | cellular amino acid metabolic process | 2 | 44 | 14.66209893 | 0.03315259 | 0.149667024 |
| GO:0043406 | positive regulation of MAP kinase activity | 2 | 44 | 14.66209893 | 0.03315259 | 0.149667024 |
| GO:0002439 | chronic inflammatory response to antigenic stimulus | 1 | 3 | 107.5220588 | 0.0370873 | 0.149667024 |
| GO:0003032 | detection of oxygen | 1 | 3 | 107.5220588 | 0.0370873 | 0.149667024 |
| GO:0003209 | cardiac atrium morphogenesis | 1 | 3 | 107.5220588 | 0.0370873 | 0.149667024 |
| GO:0007144 | female meiosis I | 1 | 3 | 107.5220588 | 0.0370873 | 0.149667024 |
| GO:0010693 | negative regulation of alkaline phosphatase activity | 1 | 3 | 107.5220588 | 0.0370873 | 0.149667024 |
| GO:0014829 | vascular smooth muscle contraction | 1 | 3 | 107.5220588 | 0.0370873 | 0.149667024 |
| GO:0014894 | response to denervation involved in regulation of muscle adaptation | 1 | 3 | 107.5220588 | 0.0370873 | 0.149667024 |
| GO:0019222 | regulation of metabolic process | 1 | 3 | 107.5220588 | 0.0370873 | 0.149667024 |
| GO:0030730 | sequestering of triglyceride | 1 | 3 | 107.5220588 | 0.0370873 | 0.149667024 |
| GO:0031401 | positive regulation of protein modification process | 1 | 3 | 107.5220588 | 0.0370873 | 0.149667024 |
| GO:0032446 | protein modification by small protein conjugation | 1 | 3 | 107.5220588 | 0.0370873 | 0.149667024 |
| GO:0032800 | receptor biosynthetic process | 1 | 3 | 107.5220588 | 0.0370873 | 0.149667024 |
| GO:0032970 | regulation of actin filament-based process | 1 | 3 | 107.5220588 | 0.0370873 | 0.149667024 |
| GO:0033314 | mitotic DNA replication checkpoint | 1 | 3 | 107.5220588 | 0.0370873 | 0.149667024 |
| GO:0034201 | response to oleic acid | 1 | 3 | 107.5220588 | 0.0370873 | 0.149667024 |
| GO:0042045 | epithelial fluid transport | 1 | 3 | 107.5220588 | 0.0370873 | 0.149667024 |
| GO:0043243 | positive regulation of protein complex disassembly | 1 | 3 | 107.5220588 | 0.0370873 | 0.149667024 |
| GO:0044320 | cellular response to leptin stimulus | 1 | 3 | 107.5220588 | 0.0370873 | 0.149667024 |
| GO:0045404 | positive regulation of interleukin-4 biosynthetic process | 1 | 3 | 107.5220588 | 0.0370873 | 0.149667024 |
| GO:0046068 | cGMP metabolic process | 1 | 3 | 107.5220588 | 0.0370873 | 0.149667024 |
| GO:0046666 | retinal cell programmed cell death | 1 | 3 | 107.5220588 | 0.0370873 | 0.149667024 |
| GO:0046668 | regulation of retinal cell programmed cell death | 1 | 3 | 107.5220588 | 0.0370873 | 0.149667024 |
| GO:0051222 | positive regulation of protein transport | 1 | 3 | 107.5220588 | 0.0370873 | 0.149667024 |
| GO:0051533 | positive regulation of NFAT protein import into nucleus | 1 | 3 | 107.5220588 | 0.0370873 | 0.149667024 |
| GO:0055118 | negative regulation of cardiac muscle contraction | 1 | 3 | 107.5220588 | 0.0370873 | 0.149667024 |
| GO:0060555 | activation of necroptosis by extracellular signals | 1 | 3 | 107.5220588 | 0.0370873 | 0.149667024 |
| GO:0060559 | positive regulation of calcidiol 1-monooxygenase activity | 1 | 3 | 107.5220588 | 0.0370873 | 0.149667024 |
| GO:0060964 | regulation of gene silencing by miRNA | 1 | 3 | 107.5220588 | 0.0370873 | 0.149667024 |
| GO:0070294 | renal sodium ion absorption | 1 | 3 | 107.5220588 | 0.0370873 | 0.149667024 |
| GO:0071476 | cellular hypotonic response | 1 | 3 | 107.5220588 | 0.0370873 | 0.149667024 |
| GO:0071930 | negative regulation of transcription involved in G1/S phase of mitotic cell cycle | 1 | 3 | 107.5220588 | 0.0370873 | 0.149667024 |
| GO:0072193 | ureter smooth muscle cell differentiation | 1 | 3 | 107.5220588 | 0.0370873 | 0.149667024 |
| GO:0086001 | regulation of cardiac muscle cell action potential | 1 | 3 | 107.5220588 | 0.0370873 | 0.149667024 |
| GO:1902042 | negative regulation of extrinsic apoptotic signaling pathway via death domain receptors | 1 | 3 | 107.5220588 | 0.0370873 | 0.149667024 |
| GO:2001274 | negative regulation of glucose import in response to insulin stimulus | 1 | 3 | 107.5220588 | 0.0370873 | 0.149667024 |
| GO:0030036 | actin cytoskeleton organization | 3 | 141 | 6.863110138 | 0.039052962 | 0.156803558 |
| GO:0044281 | small molecule metabolic process | 10 | 1363 | 2.366589703 | 0.040716327 | 0.160869295 |
| GO:0035335 | peptidyl-tyrosine dephosphorylation | 2 | 49 | 13.16596639 | 0.040799416 | 0.160869295 |
| GO:0006921 | cellular component disassembly involved in execution phase of apoptosis | 2 | 50 | 12.90264706 | 0.042413823 | 0.160869295 |
| GO:0051592 | response to calcium ion | 2 | 50 | 12.90264706 | 0.042413823 | 0.160869295 |
| GO:0006281 | DNA repair | 4 | 276 | 4.674872123 | 0.043742843 | 0.160869295 |
| GO:0044255 | cellular lipid metabolic process | 3 | 148 | 6.538503577 | 0.044508185 | 0.160869295 |
| GO:0006888 | ER to Golgi vesicle-mediated transport | 2 | 52 | 12.4063914 | 0.045726179 | 0.160869295 |
| GO:0050728 | negative regulation of inflammatory response | 2 | 52 | 12.4063914 | 0.045726179 | 0.160869295 |
| GO:0051028 | mRNA transport | 2 | 53 | 12.17230855 | 0.047423757 | 0.160869295 |
| GO:0000018 | regulation of DNA recombination | 1 | 4 | 80.64154412 | 0.049373721 | 0.160869295 |
| GO:0003383 | apical constriction | 1 | 4 | 80.64154412 | 0.049373721 | 0.160869295 |
| GO:0006497 | protein lipidation | 1 | 4 | 80.64154412 | 0.049373721 | 0.160869295 |
| GO:0007386 | compartment pattern specification | 1 | 4 | 80.64154412 | 0.049373721 | 0.160869295 |
| GO:0007412 | axon target recognition | 1 | 4 | 80.64154412 | 0.049373721 | 0.160869295 |
| GO:0009187 | cyclic nucleotide metabolic process | 1 | 4 | 80.64154412 | 0.049373721 | 0.160869295 |
| GO:0010748 | negative regulation of plasma membrane long-chain fatty acid transport | 1 | 4 | 80.64154412 | 0.049373721 | 0.160869295 |
| GO:0010757 | negative regulation of plasminogen activation | 1 | 4 | 80.64154412 | 0.049373721 | 0.160869295 |
| GO:0010950 | positive regulation of endopeptidase activity | 1 | 4 | 80.64154412 | 0.049373721 | 0.160869295 |
| GO:0014826 | vein smooth muscle contraction | 1 | 4 | 80.64154412 | 0.049373721 | 0.160869295 |
| GO:0032269 | negative regulation of cellular protein metabolic process | 1 | 4 | 80.64154412 | 0.049373721 | 0.160869295 |
| GO:0032411 | positive regulation of transporter activity | 1 | 4 | 80.64154412 | 0.049373721 | 0.160869295 |
| GO:0032914 | positive regulation of transforming growth factor beta1 production | 1 | 4 | 80.64154412 | 0.049373721 | 0.160869295 |
| GO:0033211 | adiponectin-mediated signaling pathway | 1 | 4 | 80.64154412 | 0.049373721 | 0.160869295 |
| GO:0033522 | histone H2A ubiquitination | 1 | 4 | 80.64154412 | 0.049373721 | 0.160869295 |
| GO:0034116 | positive regulation of heterotypic cell-cell adhesion | 1 | 4 | 80.64154412 | 0.049373721 | 0.160869295 |
| GO:0042759 | long-chain fatty acid biosynthetic process | 1 | 4 | 80.64154412 | 0.049373721 | 0.160869295 |
| GO:0043242 | negative regulation of protein complex disassembly | 1 | 4 | 80.64154412 | 0.049373721 | 0.160869295 |
| GO:0043631 | RNA polyadenylation | 1 | 4 | 80.64154412 | 0.049373721 | 0.160869295 |
| GO:0043949 | regulation of cAMP-mediated signaling | 1 | 4 | 80.64154412 | 0.049373721 | 0.160869295 |
| GO:0045123 | cellular extravasation | 1 | 4 | 80.64154412 | 0.049373721 | 0.160869295 |
| GO:0046323 | glucose import | 1 | 4 | 80.64154412 | 0.049373721 | 0.160869295 |
| GO:0048742 | regulation of skeletal muscle fiber development | 1 | 4 | 80.64154412 | 0.049373721 | 0.160869295 |
| GO:0050878 | regulation of body fluid levels | 1 | 4 | 80.64154412 | 0.049373721 | 0.160869295 |
| GO:0051152 | positive regulation of smooth muscle cell differentiation | 1 | 4 | 80.64154412 | 0.049373721 | 0.160869295 |
| GO:0060117 | auditory receptor cell development | 1 | 4 | 80.64154412 | 0.049373721 | 0.160869295 |
| GO:0060406 | positive regulation of penile erection | 1 | 4 | 80.64154412 | 0.049373721 | 0.160869295 |
| GO:0060742 | epithelial cell differentiation involved in prostate gland development | 1 | 4 | 80.64154412 | 0.049373721 | 0.160869295 |
| GO:0061299 | retina vasculature morphogenesis in camera-type eye | 1 | 4 | 80.64154412 | 0.049373721 | 0.160869295 |
| GO:0070265 | necrotic cell death | 1 | 4 | 80.64154412 | 0.049373721 | 0.160869295 |
| GO:0070574 | cadmium ion transmembrane transport | 1 | 4 | 80.64154412 | 0.049373721 | 0.160869295 |
| GO:0071316 | cellular response to nicotine | 1 | 4 | 80.64154412 | 0.049373721 | 0.160869295 |
| GO:0075733 | intracellular transport of viral material | 1 | 4 | 80.64154412 | 0.049373721 | 0.160869295 |
| GO:0090005 | negative regulation of establishment of protein localization to plasma membrane | 1 | 4 | 80.64154412 | 0.049373721 | 0.160869295 |
| GO:2000010 | positive regulation of protein localization to cell surface | 1 | 4 | 80.64154412 | 0.049373721 | 0.160869295 |
| GO:2000177 | regulation of neural precursor cell proliferation | 1 | 4 | 80.64154412 | 0.049373721 | 0.160869295 |
| GO:2000343 | positive regulation of chemokine (C-X-C motif) ligand 2 production | 1 | 4 | 80.64154412 | 0.049373721 | 0.160869295 |

**Supplementary Table.5 List of 42 key miRNA targets**

| **miRNA** | **Description** | **Gene_symbol** | **style** | **fold change**  **(MDS/control)** | ***P* value** | **FDR** |
| --- | --- | --- | --- | --- | --- | --- |
| miR-19a/19b/195 | acyl-CoA synthetase long-chain family member 1 | ACSL1 | down | -2.66 | 0.003 | 0.024 |
| miR-19a/19b/195 | calmodulin 1 (phosphorylase kinase, delta) | CALM1 | down | -1.87 | 0.001 | 0.005 |
| miR-19a/19b/195 | insulin-like growth factor 2 receptor | IGF2R | down | -2.91 | 0.015 | 0.073 |
| miR-19a/19b/195 | potassium inwardly-rectifying channel, subfamily J, member 2 | KCNJ2 | down | -4.29 | <0.001 | 0.001 |
| miR-642b/4497/4530 | neurexin 2 | NRXN2 | up | 3.88 | <0.001 | 0.015 |
| miR-19a/19b/125b | prosaposin | PSAP | down | -2.01 | <0.001 | 0.013 |
| miR-19a/19b/195 | solute carrier family 11 (proton-coupled divalent metal ion transporters), member 2 | SLC11A2 | down | -4.11 | <0.001 | 0.003 |
| miR-19a/19b/125b | tumor necrosis factor | TNF | down | -1.70 | 0.029 | 0.095 |
| miR-145/4423 | cyclin D2 | CCND2 | up | 1.73 | 0.002 | 0.017 |
| miR-19a/19b | endothelin receptor type B | EDNRB | down | -5.32 | <0.001 | 0.002 |
| miR-145/200c | ephrin-A1 | EFNA1 | up | 1.73 | 0.002 | 0.017 |
| miR-4530/4745 | fibroblast growth factor receptor 2 | FGFR2 | up | 2.70 | 0.009 | 0.047 |
| miR-532/145 | frizzled family receptor 7 | FZD7 | up | 2.55 | 0.015 | 0.071 |
| miR-145/148a | hematopoietically expressed homeobox | HHEX | up | 1.77 | 0.018 | 0.081 |
| miR-4505/200c | Kruppel-like factor 3 (basic) | KLF3 | up | 2.77 | 0.003 | 0.025 |
| miR-19a/19b | mastermind-like 1 (Drosophila) | MAML1 | down | -1.86 | 0.001 | 0.008 |
| miR-148a/200c | neural cell adhesion molecule 1 | NCAM1 | up | 2.08 | 0.013 | 0.067 |
| miR-17/195 | Rap guanine nucleotide exchange factor (GEF) 1 | RAPGEF1 | down | -2.77 | <0.001 | 0.005 |
| miR-19a/19b | SAR1 homolog B (S. cerevisiae) | SAR1B | down | -2.22 | <0.001 | 0.003 |
| miR-19a/19b | SEC22 vesicle trafficking protein homolog B (S. cerevisiae) (gene/pseudogene) | SEC22B | down | -2.00 | <0.001 | 0.001 |
| miR-19a/19b | solute carrier family 8 (sodium/calcium exchanger), member 1 | SLC8A1 | down | -5.63 | 0.002 | 0.017 |
| miR-17/195 | sortilin 1 | SORT1 | down | -3.06 | 0.005 | 0.035 |
| miR-19a/19b | thrombospondin 1 | THBS1 | down | -6.88 | 0.012 | 0.063 |
| miR-19a/19b | tumor necrosis factor receptor superfamily, member 1B | TNFRSF1B | down | -4.78 | <0.001 | 0.007 |
| miR-195 | brain-derived neurotrophic factor | BDNF | down | -1.96 | <0.001 | 0.002 |
| miR-17 | calcium/calmodulin-dependent protein kinase II delta | CAMK2D | down | -2.16 | <0.001 | <0.001 |
| miR-195 | cell division cycle 25 homolog B (S. pombe) | CDC25B | down | -1.93 | 0.003 | 0.027 |
| miR-195 | delta-like 1 (Drosophila) | DLL1 | down | -5.49 | <0.001 | 0.005 |
| miR-4505 | EH-domain containing 2 | EHD2 | up | 1.84 | 0.005 | 0.066 |
| miR-4530 | F11 receptor | F11R | up | 1.85 | 0.025 | 0.099 |
| miR-145 | filamin B, beta | FLNB | up | 1.61 | 0.028 | 0.087 |
| miR-148a | high mobility group AT-hook 2 | HMGA2 | down | -2.26 | <0.001 | <0.001 |
| miR-195 | interleukin 10 receptor, alpha | IL10RA | down | -2.62 | 0.014 | 0.068 |
| miR-148a | integrin, alpha 9 | ITGA9 | up | 3.05 | 0.0024 | 0.032 |
| miR-148a | v-kit Hardy-Zuckerman 4 feline sarcoma viral oncogene homolog | KIT | up | 1.70 | 0.024 | 0.041 |
| miR-145 | Meis homeobox 1 | MEIS1 | up | 2.42 | 0.026 | 0.102 |
| miR-195 | muscle RAS oncogene homolog | MRAS | down | -3.33 | 0.001 | 0.013 |
| miR-195 | myosin light chain kinase | MYLK | down | -3.74 | 0.001 | 0.012 |
| miR-4530 | placental growth factor | PGF | up | 1.53 | 0.018 | 0.082 |
| miR-17/122/4668 | sprouty homolog 1, antagonist of FGF signaling (Drosophila) | SPRY1 | up | 3.09 | 0.002 | 0.021 |
| miR-122 | sprouty homolog 2 (Drosophila) | SPRY2 | up | 2.61 | 0.002 | 0.019 |
| miR-148a | TEK tyrosine kinase, endothelial | TEK | up | 3.30 | 0.004 | 0.031 |

**Supplementary Table.6 Primer sequences for quantification and cloning.**

| **Gene** | **F/R** | **Sequence** |
| --- | --- | --- |
| **RT-PCR** |  |  |
| miR-195-5p | F | Cat.No CD201-0083, Tiangan, Beijing, China |
|  | R |  |
| miR-19a-3p | F | Cat.No CD201-0021, Tiangan, Beijing, China |
|  | R |  |
| miR-17-3p | F | Cat.No CD201-0017, Tiangan, Beijing, China |
|  | R |  |
| U6 | F | 5’-CTTCGGCAGCACATATACT-3’ |
|  | R | 5’-AAAATATGGAACGCTTCACG-3’ |
| DLL1 | F | 5’-GATTCTCCTGATGACCTCGCA-3’ |
|  | R | 5’-TCCGTAGTAGTGTTCGTCACA-3’ |
| MAML1 | F | 5’-CCCCAGTGAGTCATTTCCTCT-3’ |
|  | R | 5’-GAGGTTGCTTTGCGATATGGA-3’ |
| CAMK2D | F | 5'-GTCACTGAACAACTGATCGAAGC-3' |
|  | R | 5'-GAATCGGTGAAAATCCATCCCTT-3' |
| GAPDH | F | 5’-GCACCGTCAAGGCTGAGAAC-3' |
|  | R | 5‘-GTGGTGAAGACGCCAGTGGA-3' |
| miR-195 cloning | F | GGAAAGGACGAAACACCGGATCTCCAGGGCAGTTTCAAG |
|  | R | TGTCTCGAGGTCGAGAATTAAAAAACGTGCTGTCTGCTTAACATTA |

**Supplementary Table.7 The expression of miRNAs and targeted genes in 36 MDS patients and 24 normal controls.**

| **No.** | **Diag** | **Sex** | **Age** | **Blast** | **Hyperplasity** | **Chr** | **CD34+ (%)** | **miR-195** | **miR-17** | **miR-19a** | **CAMK2D** | **MAML1** | **DLL1** |
| --- | --- | --- | --- | --- | --- | --- | --- | --- | --- | --- | --- | --- | --- |
| MP1 | RAEB-1 | F | 62 | 5.0 | hyper | del(9q13q22),del(20q12) | 3.7 | 2.2 | 1.3 | 4.7 | 0.2 | 0.1 | 1.2 |
| MP2 | RCMD | M | 45 | 3.0 | hyper | tri8 | 1.4 | 6.4 | 14.4 | 2.6 | 0.6 | 0.2 | 0.1 |
| MP3 | RCMD | F | 40 | 0.4 | hyper | tri8 | 0.4 | 2.8 | 0.2 | 10.5 | 1.0 | 0.2 | 0.3 |
| MP4 | RCMD | M | 81 | 3.6 | hyper | tri8 | 7.0 | 2.7 | 2.0 | 9.7 | 0.3 | 0.1 | 0.4 |
| MP5 | RAEB-1 | M | 68 | 8.6 | hyper | mono3, del(5q12q31), mono6,tri8 | 7.4 | 2.6 | 125.5 | 1.5 | 0.5 | 0.6 | 0.0 |
| MP6 | RCMD | M | 29 | 1.2 | hypo | tri8 | 0.6 | 5.6 | 26.8 | 0.7 | 1.2 | 1.3 | 0.2 |
| MP7 | RCMD | M | 57 | 1.0 | hypo | tri8 | 1.8 | 28.2 | 7.0 | 0.5 | 1.6 | 1.5 | 0.7 |
| MP8 | RAEB-1 | F | 60 | 5.5 | hyper | normal | 6.5 | 1.0 | 1.5 | 28.0 | 2.3 | 0.0 | 0.1 |
| MP9 | RAEB-2 | M | 68 | 10.5 | hyper | der(3;5)(q10;p10),mono6,tri8 | 11.7 | 2.2 | 1.7 | 60.5 | 0.1 | 0.0 | 1.2 |
| MP10 | RAEB-2 | F | 64 | 10.0 | hyper | mono7 | 5.1 | 1.0 | 1.0 | 26.8 | 2.4 | 0.3 | 0.3 |
| MP11 | RCMD | F | 62 | 2.0 | hyper | normal | 1.4 | 2.0 | 4.3 | 2.6 | 0.2 | 0.2 | 1.1 |
| MP12 | RCMD | M | 60 | 1.0 | hyper | der(5)t(5;11)(q31;q13),del(17p11.2) | 6.9 | 0.6 | 28.0 | 1.3 | 0.6 | 0.4 | 0.0 |
| MP13 | RAEB-1 | F | 59 | 6.4 | hyper | normal | 6.1 | 1.8 | 0.6 | 31.6 | 0.5 | 0.2 | 0.4 |
| MP14 | RAEB-1 | M | 61 | 6.5 | hyper | normal | 2.9 | 1.9 | 0.8 | 5.7 | 0.1 | 0.6 | 1.0 |
| MP15 | RCMD | F | 58 | 1.0 | hyper | tri8 | 1.1 | 1.9 | 3.6 | 0.6 | 0.1 | 0.4 | 1.2 |
| MP16 | RCMD | M | 52. | 3.0 | hyper | del(5q),-7,-12,-13,-18 | 3.9 | 1.7 | 2.8 | 8.4 | 1.4 | 0.1 | 0.9 |
| MP17 | RAEB-1 | M | 70 | 5.0 | hyper | tri8 | 8.7 | 1.6 | 4.7 | 2.4 | 0.2 | 0.3 | 0.7 |
| MP18 | RCMD | M | 56 | 1.2 | hypo | NA | 0.3 | 1.3 | 0.8 | 25.3 | 3.4 | 0.1 | 0.4 |
| MP19 | RAEB-2 | F | 64 | 11.0 | hyper | normal | 12.1 | 4.0 | 2.6 | 18.2 | 0.1 | 0.1 | 0.3 |
| MP20 | RAEB-2 | M | 50 | 10.5 | hyper | normal | 2.8 | 2.4 | 0.7 | 17.0 | 0.3 | 0.0 | 0.4 |
| MP21 | RAEB-2 | F | 61 | 14.4 | hyper | del5q- | 15.4 | 1.5 | 2.1 | 5.4 | 2.6 | 0.5 | 1.2 |
| MP22 | RCMD | M | 44 | 0.2 | hypo | normal | 0.1 | 3.0 | 4.8 | 7.0 | 1.6 | 0.1 | 0.3 |
| MP23 | RCMD | M | 56 | 0.4 | hypo | tri8 | 1.1 | 22.3 | 2.9 | 16.7 | 0.7 | 0.3 | 0.5 |
| MP23 | RCMD | M | 56 | 0.4 | hypo | tri8 | 1.1 | 0.6 | 2.9 | 16.7 | 0.7 | 0.3 | 0.5 |
| MP24 | RCMD | F | 51 | 3.0 | hyper | normal | 2.4 | 1.2 | 5.4 | 3.6 | 0.2 | 1.0 | 0.7 |
| MP25 | RCMD | F | 64 | 0.8 | hyper | complex | 2.0 | 0.6 | 1.3 | 5.4 | 0.2 | 0.5 | 0.9 |
| MP26 | RCMD | M | 53 | 2.8 | hyper | normal | 2.3 | 1.9 | 0.5 | 7.2 | 2.6 | 0.5 | 0.9 |
| MP27 | RAEB-1 | F | 40 | 9.2 | hyper | normal | 4.4 | 1.4 | 1.5 | 6.8 | 5.4 | 0.7 | 0.3 |
| MP28 | RAEB-1 | F | 40 | 4.6 | hyper | normal | 6.5 | 0.4 | 9.0 | 20.6 | 0.1 | 0.1 | 0.5 |
| MP29 | RAEB-1 | M | 78 | 5.4 | hyper | del5q-,inv9 | 4.1 | 5.8 | 1.9 | 30.4 | 0.8 | 0.1 | 0.1 |
| MP30 | RAEB-1 | M | 79 | 6.2 | hyper | complex | 6.2 | 1.7 | 1.4 | 1.8 | 1.7 | 0.2 | 0.9 |
| MP31 | RAEB-2 | F | 39 | 15.2 | hyper | complex | 6.3 | 0.3 | 4.9 | 0.4 | 0.5 | 2.6 | 3.1 |
| MP32 | RCMD | M | 72 | 1.2 | hypo | normal | 1.3 | 5.4 | 0.8 | 0.3 | 0.9 | 2.1 | 0.1 |
| MP33 | RAEB-2 | F | 70 | 11.6 | hyper | normal | 3.7 | 1.7 | 3.3 | 1.5 | 4.7 | 0.6 | 0.4 |
| MP34 | RAEB-2 | F | 34 | 25.0 | hyper | normal | 9.8 | 3.1 | 17.0 | 1.5 | 0.2 | 3.1 | 0.4 |
| MP35 | RCMD | M | 46 | 0.2 | hyper | tri8 | 0.4 | 18.6 | 0.4 | 4.8 | 3.2 | 0.5 | 0.0 |
| MDSL | RAEB-2 | M | NA | 15.2 | hyper | complex | 27.9 | 1.5 | 4.6 | 2.9 | 1.0 | 0.7 | 1.3 |
| HC1 | NC | F | 29 | NA | NA | NA | NA | 1.4 | 0.7 | 1.6 | 1.5 | 2.6 | 1.5 |
| HC2 | NC | F | 51 | NA | NA | NA | NA | 0.7 | 0.3 | 0.5 | 0.6 | 3.4 | 2.3 |
| HC3 | NC | M | 28 | NA | NA | NA | NA | 0.9 | 2.1 | 1.7 | 2.4 | 0.6 | 1.1 |
| HC4 | NC | M | 46 | NA | NA | NA | NA | 4.5 | 0.7 | 2.5 | 3.5 | 0.6 | 1.0 |
| HC5 | NC | F | 91 | NA | NA | NA | NA | 0.4 | 0.4 | 3.8 | 6.1 | 0.5 | 0.6 |
| HC6 | NC | M | 62 | NA | NA | NA | NA | 0.9 | 0.5 | 6.5 | 3.2 | 0.1 | 2.4 |
| HC7 | NC | M | 74 | NA | NA | NA | NA | 0.7 | 2.3 | 7.9 | 0.6 | 0.2 | 2.3 |
| HC8 | NC | F | 51 | NA | NA | NA | NA | 0.5 | 1.2 | 0.4 | 0.3 | 0.6 | 3.1 |
| HC9 | NC | M | 58 | NA | NA | NA | NA | 3.2 | 1.8 | 0.1 | 0.1 | 0.1 | 0.3 |
| HC10 | NC | M | 59 | NA | NA | NA | NA | 0.7 | 1.4 | 0.1 | 0.1 | 0.1 | 0.4 |
| HC11 | NC | M | 63 | NA | NA | NA | NA | 2.1 | 2.0 | 1.2 | 0.3 | 0.3 | 0.4 |
| HC12 | NC | M | 60 | NA | NA | NA | NA | 1.3 | 2.8 | 0.6 | 0.6 | 0.1 | 0.3 |
| HC13 | NC | F | 61 | NA | NA | NA | NA | 0.7 | 1.0 | 0.6 | 1.2 | 0.9 | 0.9 |
| HC14 | NC | F | 74 | NA | NA | NA | NA | 2.1 | 0.5 | 0.4 | 1.1 | 0.1 | 0.7 |
| HC15 | NC | M | 82 | NA | NA | NA | NA | 1.2 | 0.7 | 0.1 | 0.9 | 1.5 | 0.2 |
| HC16 | NC | F | 35 | NA | NA | NA | NA | 1.8 | 1.3 | 0.3 | 1.0 | 2.4 | 0.2 |
| HC17 | NC | M | 44 | NA | NA | NA | NA | 3.5 | 0.9 | 0.3 | 2.4 | 2.8 | 1.3 |
| HC18 | NC | F | 48 | NA | NA | NA | NA | 2.5 | 0.3 | 0.6 | 1.8 | 0.3 | 0.7 |
| HC19 | NC | F | 49 | NA | NA | NA | NA | 4.6 | 0.5 | 0.9 | 0.1 | 0.2 | 1.8 |
| HC20 | NC | F | 53 | NA | NA | NA | NA | 6.5 | 0.7 | 0.4 | 0.0 | 5.1 | 0.8 |
| HC21 | NC | M | 67 | NA | NA | NA | NA | 0.5 | 0.5 | 0.6 | 0.4 | 1.6 | 1.7 |
| HC22 | NC | M | 19 | NA | NA | NA | NA | 0.3 | 0.3 | 0.2 | 0.0 | 0.1 | 0.5 |
| HC23 | NC | F | 27 | NA | NA | NA | NA | 0.5 | 0.5 | 0.2 | 0.2 | 0.4 | 0.7 |
| HC24 | NC | F | 36 | NA | NA | NA | NA | 0.6 | 0.6 | 0.1 | 0.4 | 0.4 | 0.4 |

NA, not available; F, female; M,male; NC, normal control
